# Collapsing the Bottleneck by Interfacial Effect of Ni/CeO_2_ for Long‐Term Hydrogen Production using Waste Alkaline Water in Practical‐Scale Anion Exchange Membrane Water Electrolyzer

**DOI:** 10.1002/advs.202502484

**Published:** 2025-06-09

**Authors:** Nam In Kim, Jaehun Lee, Song Jin, Jaehoon Jeong, Shin‐Woo Myeong, Jun Seok Ha, Junyoung Park, Hoseok Lee, Minjeong Park, Chiho Kim, Sungjun Kim, Seok Hwan Yang, Yoo Sei Park, Jooyoung Lee, Jang Yong Lee, Min Ho Seo, Sung Mook Choi

**Affiliations:** ^1^ Energy& Environment Materials Research Division Korea Institute of Materials Science (KIMS) Changwon 51508 Republic of Korea; ^2^ Department of Materials Science and Engineering Pusan National University Busan 46241 Republic of Korea; ^3^ School of Materials Science and Engineering Gwangju Institute of Science and Technology (GIST) 261 Cheomdan‐gwagiro Gwangju 500‐712 Republic of Korea; ^4^ Hydrogen Energy Research Center Korea Research Institute of Chemical Technology Daejeon 34114 Republic of Korea; ^5^ Department of Nanoenergy Engineering Pusan National University 50, Busandaehak‐ro 63 beon‐gil 2, Geumjeong‐gu Busan 46241 Republic of Korea; ^6^ Department of Nano Fusion Technology Pusan National University Busandaehak‐ro 63 beon‐gil 2, Geumjeong‐gu Busan 46241 Republic of Korea; ^7^ Department of Chemical Engineering Konkuk University Seoul 05029 Republic of Korea; ^8^ Department of Nanotechnology Engineering Pukyong National University 45 Yongso‐ro, Nam‐gu Busan 48547 Republic of Korea; ^9^ Advanced Materials Engineering University of Science and Technology (UST) Daejeon 34113 Republic of Korea

**Keywords:** AEMWE, EMSI effect, hydrogen evolution reaction, non‐precious metal electrocatalysts, practical scale hydrogen production, waste alkaline water electrolysis

## Abstract

The demand for hydrogen production compels the development of various strategies for water splitting. Among these strategies, the Anion Exchange Membrane Water Electrolyzer (AEMWE) offers the advantages such as low cost and the production of high‐purity hydrogen. Waste alkaline water generated from various industries can be directly used in the AEMWE system, due to its appropriate pH range of 13–14. While various nickel‐based hydrogen evolution reaction (HER) electrocatalysts have demonstrated adequate performance, their long‐term stability remains a concern. To ensure long‐term stability of AEMWE, it is crucial to address the potential poisoning effects of impurities present in waste alkaline water on nickel‐based HER electrocatalysts. In this study, the Ni‐CeO₂/Carbon (NCC) catalyst is employed for HER in waste alkaline water containing Li, Mo, and W ions. NCC exhibited an overpotential of 31 mV, showing ≈3 times less overpotential compared to the Ni/C. The XPS results and DFT calculations demonstrated the electrons transferring from CeO_2_ to Ni, inducing the Electronic Metal‐Support Interactions (EMSI) effects. The NCC shows 1 A cm^−2^ at 2.03 V in AEMWE while Ni/C needed 2.33 V to achieve the same current densities. The NCC maintaines long‐term stability over 2000 h, with a degradation rate of 4.95%.

## Graphical abstract

The practical scale of AEMWE is achieved using waste alkaline water and non‐precious metals for electrolytes and electrocatalysts. Electrocatalysts incorporating Ni with CeO₂ demonstrate EMSI effects, boosting both performance and durability. The AEMWE system, with an active area of 64 cm^2^, undergoes a 2000‐h durability test, resulting in a low degradation rate of 4.95%.

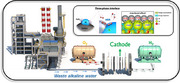

## 1 Introduction

Wastewater with high alkalinity generated by various industrial processes has contamination issues, necessitating purification.^[^ ^1^ ^]^ Purifying this waste alkaline water presents significant cost challenges, potentially hindering the commercial scaling of electrolysis.^[^ ^2^ ^]^ Waste alkaline water, with a pH value of ≈14, remains great challenging for alkaline water electrolysis (AWE) due to its high concentration of electrolytes (≈4.5–6.5 m KOH), meaning the low concentration of waste alkaline water (1.0 m KOH) cannot be directly used as an electrolyte.^[^ ^3^ ^]^ The proton exchange membrane water electrolysis (PEMWE) system operates in an acidic environment, making it unsuitable for directly using waste alkaline water as an electrolyte. Additionally, both AWE and PEMWE, which require high‐purity electrolytes, are not suitable for using wastewater, which includes various impurities.^[^ ^2^, ^4^ ^]^ In contrast, Anion Exchange Membrane Water Electrolysis (AEMWE) operates effectively at pH levels of ≈13–14, allowing for the direct application of waste alkaline water as an electrolyte, thus providing a viable recycling pathway. Furthermore, non‐precious materials, or significantly reduced quantities of precious materials, can serve as electrocatalysts for both the hydrogen evolution reaction (HER) and the oxygen evolution reaction (OER), providing a cost‐effective approach.^[^ ^5^ ^]^ However, the presence of impurity ions in waste alkaline water may adhere to the electrode. This adsorption can result in poisoning effects, which could potentially compromise the electrocatalytic performance.^[^ ^6^ ^]^ Specifically, the adsorption of cations from the wastewater onto cathode materials demands strict attention to the effective strategies for designing HER electrocatalysts to enhance long‐term AEMWE system performance.

In alkaline media, the hydrogen evolution reaction (HER) occurs via a two‐step process. Initially, water (H_2_O), serving as the proton donor, dissociates into adsorbed hydrogen (H_ads_) and hydroxide ions (OH⁻) in what is referred to as the Volmer step. The adsorbed hydrogen can subsequently participate in either the Tafel or Heyrovsky step.^[^ ^7^ ^]^ It is important to note that the alkaline Volmer step, which involves the dissociation of strong covalent bonds in H_2_O to generate H_(ads)_ on active sites, requires more energy compared to the acidic Volmer step, which produces H_(ads)_ more readily from hydronium ions (H_3_O^+^).^[^ ^8^ ^]^

The Strong Metal‐Support Interactions (SMSI) have been extensively studied since the interaction between noble metals and titanium dioxide (TiO_2_) was first investigated.^[^ ^9^ ^]^ To further elucidate the enhancement of catalytic properties, the concept of Electronic Metal‐Support Interactions (EMSI) was introduced.^[^ ^10^ ^]^ The EMSI effect has been applied in various fields, contributing to improvements in both the catalytic activity and stability of catalysts.^[^ ^11^ ^]^ Various strategies, including hetero‐metal atom integration, surface ligand modification, and tuning of electronic metal‐support interaction (EMSI), have been explored to develop efficient electrocatalysts. Among them, EMSI has been reported to be effective in decoupling the relationship between ΔGH^*^ and HER activity by modulating the d‐band center through orbital rehybridization and charge redistribution. Moreover, EMSI enables hydrogen spillover from the metal to the support, further enhancing the HER kinetics.^[^ ^12^ ^]^ Among the various transition metal oxides, CeO_2_, which contains numerous oxygen vacancies, is a well‐known support for interacting with transition metals.^[^ ^13^ ^]^ This interaction facilitates the EMSI effect, thereby enhancing the catalytic properties.^[^ ^11^, ^14^ ^]^

In this study, we strategically designed and developed the heterostructure electrocatalyst, composed of Ni and CeO_2_ on a carbon substrate (NCC), to achieve enhanced performance in the HER. The Ni and CeO_2_ were grown on the carbon substrate using an island‐type growth. The incorporation of Ni into the CeO_2_ structure resulted in significant modifications to the electronic structures of Ni, attributed to electron transfer from CeO_2_. This electron transfer, as demonstrated by DFT calculations, established strong chemical bonding between Ni and CeO_2_, enhancing the catalyst's durability through EMSI effects. Furthermore, the NCC for HER electrocatalyst was employed in an AEMWE system designed for practical‐scale applications, with an active surface area of 64 cm^2^. The designed AEMWE was operated in lithium‐rich waste alkaline water (Li, Mo, W) with a pH of 13.4. The AEMWE system demonstrated voltages of 1.83 V and 2.03 V at current densities of 0.5 and 1 A cm^−2^, respectively. In addition, the cell efficiency was exhibited to 72.79%, with an energy consumption of 45.86 kWh kg^−1^. Notably, the long‐term durability analysis demonstrated that at a current density of 550 mA cm^−2^ exhibited a degradation rate of only 4.95% over a duration of 2000 h.

## 2 Results and Discussion

### 2.1 Synthesis and Physicochemical Analysis

**Figure 1:**
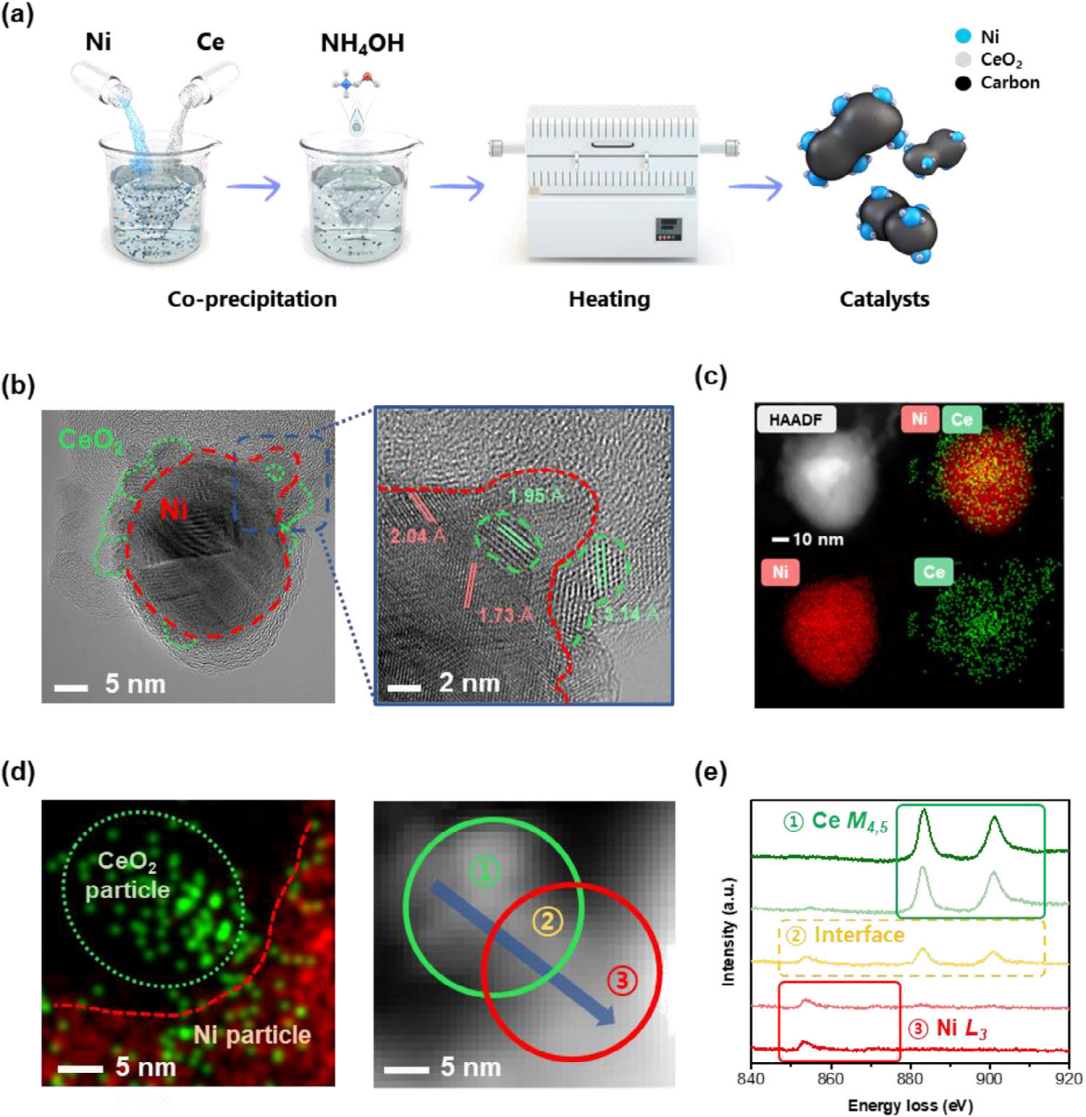
The synthesis and surface characterization of NCC. a) Synthesis and structural characterizations of NCC. b) HR‐TEM image of NCC and the magnification image for the interface between Ni and CeO_2_. c) HAADF and EDS mapping images of NCC at the same region of the HR‐TEM image. d) HAADF mapping and EELS images of the interface of Ni and CeO_2_. e) The line scanning EELS spectra including Ni, CeO_2_, and the interface of Ni and CeO_2_.

By directly employing waste alkaline water, the critical issues such as additional demands of power and energy for purifying the water in AEMWE can be alleviated. To demonstrate the improvement in both performance and durability of AEMWE in a waste alkaline water environment, the NCC, composed of non‐noble metals, was synthesized as illustrated in the scheme (**Figure** **1**a). Ni and Ce ions were precipitated onto the carbon, followed by heat treatment at 500 °C in a H_2_(4%)/N_2_ atmosphere, resulting in a reduction process. The surface morphology of the synthesized electrocatalysts was investigated using High‐Resolution Transmission Electron Microscopy (HR‐TEM) (Figure 1b). The particle size of CeO_2_ in the NCC composite was investigated to be ≈3.5 nm, while the particle size of Ni was ≈20.5 nm (Figure S1, Supporting Information). Most of the CeO_2_ was observed adjacent to the Ni particles, indicating a close interaction and functionalization between CeO_2_ and Ni could be possible within the interfacial heterostructure. The distinctive interplanar spacing (d‐spacing) values of Ni and CeO_2_ were confirmed using Selected Area Electron Diffraction (SAED) patterns, which were calculated via Fast Fourier Transform (FFT) (Figure S2, Supporting Information). The d‐spacing values of 3.14 and 1.95 Å for CeO_2_ and 2.04 and 1.73 Å for Ni were consistent with the results obtained from the SAED pattern. To examine the atomic composition of NCC, High Angle Annular Dark Field (HAADF) and atom mapping analysis were conducted (Figure 1c). It revealed that CeO_2_ particles were located adjacent to the Ni surfaces. Furthermore, Ni was uniformly distributed on the carbon substrate, with the majority of CeO_2_ particles positioned alongside Ni (Figure S3, Supporting Information).

To investigate the interface between Ni and CeO_2_ in NCC, Electron Energy Loss Spectroscopy (EELS) analysis was conducted.^[^ ^15^ ^]^ The interfacial surface between Ni and CeO_2_ was determined using the HAADF mapping image of NCC (Figure 1d). The EELS results were obtained via line scanning, including the area of Ni, CeO_2,_ and the interface (Figure 1e). The results of CeO_2_ and Ni were determined through the region of Ce M_4,5_ and Ni L_3_, respectively. The EELS analysis demonstrated the co‐existence of Ni and CeO_2_ at the interface, indicating the close interaction between the two materials.

**Figure 2:**
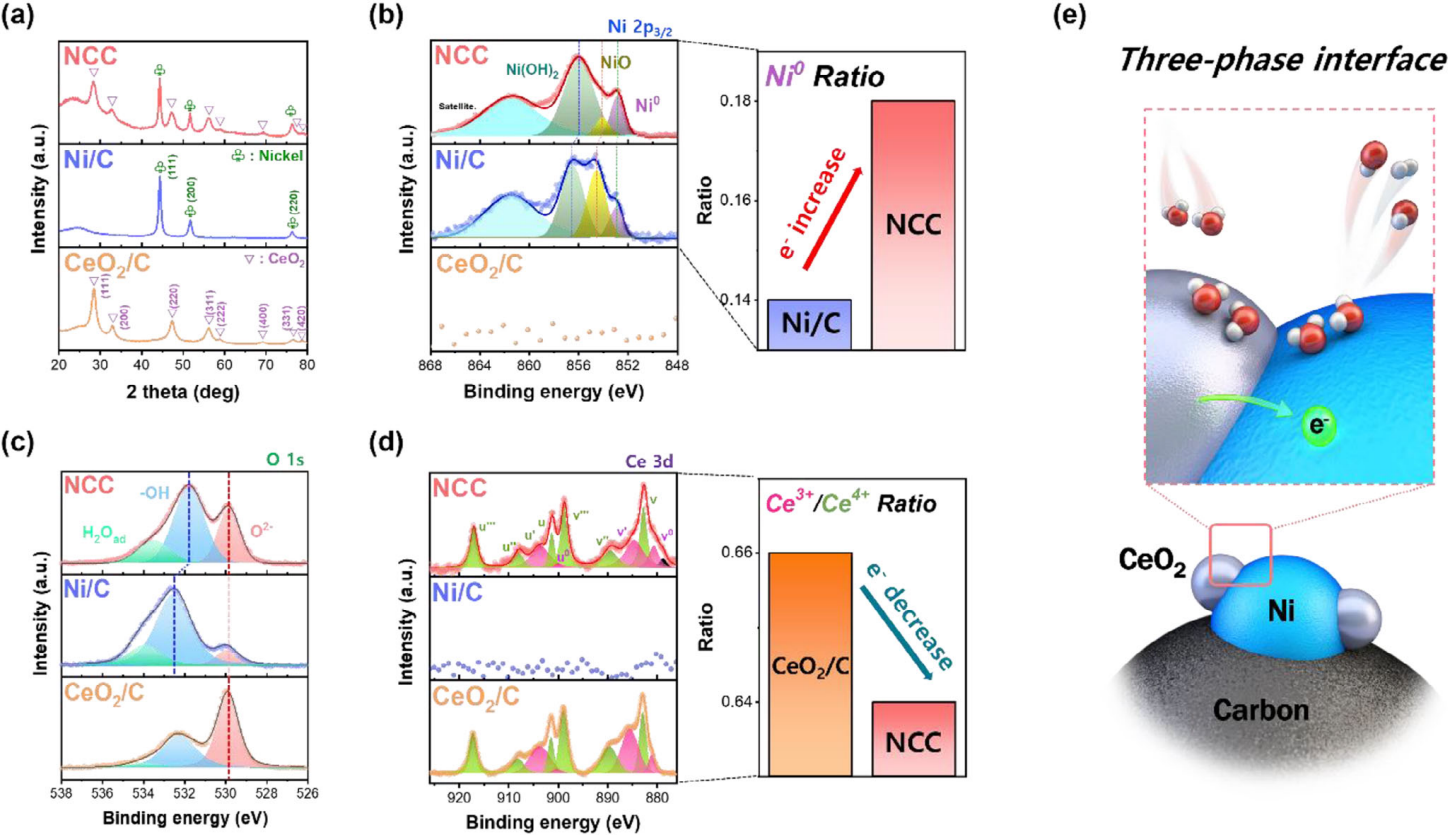
The physical and electronic structure of NCC, Ni/C, and CeO_2_/C. a) XRD patterns of NCC, Ni/C, and CeO_2_/C. XPS results of NCC, Ni/C, and CeO_2_/C were shown. b) The Ni 2p_3/2_ peak with 3 different peaks and the ratio plot of Ni^0^/Ni^2+^. c) The O 1s peak plot with 3 different peaks. d) The Ce 3d peak was separated into 10 different peaks, and the ratio plot of Ce^3+^/Ce^4+^. e) The scheme of NCC and the interface of Ni and CeO_2_.

For detailed structure analysis for the NCC, X‐ray diffraction (XRD) analysis was performed (**Figure** **2**a). Ni/C exhibited 2‐theta peaks at 44.4, 51.7, and 76.3°, corresponding to the diffraction planes (111), (200), and (220), respectively (JCDPS No. 04‐0850). CeO_2_ was diffracted with four main peaks at 2‐theta angles of 28.5, 32.8, 47.3, and 56.1°, corresponding to diffraction planes (111), (200), (220), and (311), respectively (JCPDS No. 34‐0394). NCC showed the 2‐theta peaks consistent with both Ni/C and CeO_2_/C, suggesting successful precipitation of Ni and CeO_2_. Inductively Coupled Plasma Optical Emission Spectroscopy (ICP‐OES) was conducted to determine the weight percentages of Ni and Ce. It revealed that Ni and Ce comprised 34.4% and 16.6% of the total weight, respectively (Figure S4, Supporting Information).

X‐ray Photoelectron Spectroscopy (XPS) analysis was conducted to evaluate the effect of CeO_2_ on the electronic structure of Ni. The Ni 2p_3/2_ peak was deconvoluted into three different peaks, Ni^0^, NiO, and Ni(OH)_2_ (Figure 2b).^[^ ^16^ ^]^ The Ni reduced in the atmosphere of H_2_/N_2_ had a thin oxide layer, resulting in the presence of NiO or Ni(OH)_2_ in XPS, which is surface analysis.^[^ ^17^ ^]^ In Ni/C, the binding energy of Ni^0^ was measured at 852.88 eV, whereas in NCC, it corresponded to 852.82 eV, showing a slight decrement between NCC and Ni/C. For NiO, Ni/C exhibited a binding energy of 854.52 eV, whereas NCC displayed a value of 854.03 eV Ni(OH)_2_ in Ni/C was recorded at 856.48 eV, while in NCC, it was investigated as 855.94 eV. Notably, NiO and Ni(OH)_2_ peaks in NCC exhibited negative shifts compared to Ni/C. Furthermore, the ratio of Ni^0^/Ni was calculated to be 0.14 in Ni/C, while in NCC, it was 0.18, showing an increment of Ni^0^. Additionally, the Ni 2p_1/2_ peaks of NCC and Ni/C showed similar tendencies with Ni 2p_3/2_ peaks (Figure S5, Supporting Information). The O 1s peak was deconvoluted into three distinct peaks representing oxygen within the lattice, hydroxyl groups, and adsorbed water molecules (Figure 2c).^[^ ^16^, ^18^ ^]^ The hydroxyl peak with the highest area ratio among the three peaks was identified at 532.63 eV in Ni/C. Conversely, CeO_2_/C displayed a lattice oxygen peak at 529.90 eV, exhibiting the highest area ratio among the three peaks. In NCC, both lattice oxygen and hydroxyl peaks were observed, originating from the presence of CeO_2_ and Ni. Specifically, the hydroxyl peak of NCC was measured at 531.77 eV, indicating a decrement of ≈0.86 eV compared to Ni/C, while the lattice oxygen peak was demonstrated as 529.84 eV, showing little change. The Ce 3d peak, deconvoluted into ten different peaks, was depicted in Figure 2d.^[^ ^19^ ^]^ Due to the presence of multiple oxygen vacancies in CeO_2_, Ce exhibited oxidation states of both +3 and +4.^[^ ^19^, ^20^ ^]^ The values of v^0^, v', u^0^, and u' indicate Ce^3+^, while the values of v, v'', v''', u, u'', and u''' correspond to Ce^4+^. The ratio of Ce^3+^/Ce^4+^ was calculated for the CeO_2_/C and NCC. The ratio of NCC and CeO_2_/C was investigated as 0.64 and 0.66. The decrement of Ce^3+^/Ce^4+^ indicated that the electron depletion occurred at CeO_2_ in NCC.^[^ ^21^ ^]^ Additionally, the significant peak shifts of both Ce^3+^ and Ce^4+^ when comparing NCC and Ni/C were not observed.

The possibility of interaction between Ni and CeO_2_ was already discussed through the EELS analysis. Based on the EELS and XPS results, it was observed that NCC exhibited a distinct electronic structure compared to the individual components of Ni and CeO_2_, as depicted in Figure 2e. Specifically, the binding energy of Ni in NCC showed negative shifts in comparison to Ni/C, accompanied by an increased intensity ratio of Ni^0^, suggesting a significant electron‐abundant situation of NCC. In the NCC electrocatalysts, although the binding energy of Ce in both NCC and CeO_2_/C did not exhibit significant shifts, there was a reduction in the intensity ratio of Ce^3+^/Ce^4+^ compared to CeO_2_/C. This reduction indicates the oxidation of CeO_2_ within the NCC composite. Furthermore, the ─OH peak of Ni in Ni/C had a negative shift by the electron transferring, which is coincident the results from the Ni2p_3/2_.

### 2.2 Electrochemical Properties

**Figure 3:**
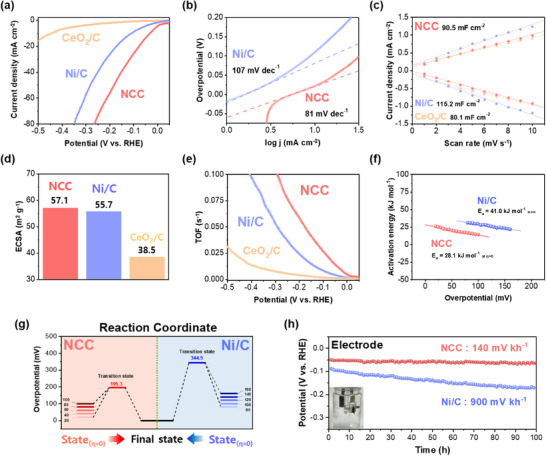
Electrochemical properties of NCC, Ni/C, and CeO_2_/C demonstrated by half‐cell analysis. a) LSV curves of NCC, Ni/C, and CeO_2_/C were analyzed. b) The Tafel slopes of NCC and Ni/C. c) The capacitance of the double layer, d) the calculated ECSA values, and e) the calculated TOF values of NCC, Ni/C, and CeO_2_/C. f) The activation energy of NCC and Ni/C. g) The reaction coordinate, including the transition state of NCC and Ni/C. h) Chronopotentiometry (C.P) analysis for investigating the durability of NCC and Ni/C.

To assess the electrocatalytic properties of NCC, Linear Sweep Voltammetry (LSV) curves were investigated with the 3‐electrode system in the waste alkaline electrolyte (**Figure** **3**a). The composition of waste alkaline water electrolyte, which is used in this study, involved various ions such as Li, W, and Mo was compared with 1 m KOH (Figure S6, Supporting Information). In the evaluation of catalytic activity, NCC showed the overpotential of 31 mV at −10 mA cm^−2^. The overpotential of CeO_2_/C, however, was 443 mV, attributed to the low catalytic activity of CeO_2_/C from the low conductivity of metal oxides compared to metallic materials.^[^ ^22^ ^]^ Ni/C exhibited an overpotential of 110 mV, having a lower overpotential than CeO_2_/C, due to the higher catalytic activity of metallic Ni. Attributable to the interfacial heterostructure between Ni and CeO_2_, NCC demonstrated significantly enhanced electrochemical properties for hydrogen production compared to the individual components, Ni and CeO₂. To facilitate a comparative analysis of the kinetic behavior exhibited by each catalyst, Tafel slopes were determined based on the LSV results (Figure 3b). The Tafel slope of NCC was 81 mV dec^−1^, while the Tafel slopes recorded for Ni/C and CeO_2_/C were 107 and 249 mV dec^−1^, respectively (Figure S7, Supporting Information). When comparing the catalytic activity of NCC in 1 m KOH with that in waste alkaline water, it was observed that NCC exhibited an overpotential of 49 mV in 1 m KOH (Figure S8, Supporting Information). To demonstrate that no side reactions occurred in the waste alkaline water, involving undesired ions such as Li, W, and Mo, the volumes of H_2_ and O_2_ were directly measured using an H‐cell (Figure S9 and Table S1, Supporting Information). The theoretical values were calculated using the method outlined in the experimental section. The volumes of H_2_ and O_2_ produced in the waste alkaline water were consistent with the results obtained from the 1 m KOH solution and showed little difference from the theoretical values.

To facilitate a comparison between the HER catalytic activity and the Electrochemical Surface Area (ECSA), the double‐layer capacitance (C_dl_) was investigated. Cyclic voltammetry (CV) analysis was conducted in the non‐faradaic region to eliminate the disturbance of the faradaic reaction (Figure S10, Supporting Information). The C_dl_ values for Ni/C and CeO_2_/C were determined to be 115.2 and 80.1 mF cm^−2^, respectively, while NCC had 90.5 mF cm^−2^ (Figure 3c). For an accurate comparison, both anodic and cathodic currents were examined, and the difference between the two values was less than 4%. The ECSA was determined using a specific capacitance (C_s_) value of 40 µF cm^−2^ (Figure 3d).^[^ ^23^ ^]^ The ECSA values for Ni/C, NCC, and CeO_2_/C were calculated to be 57.3, 55.7, and 38.5 m^2^ g^−1^, respectively. Despite the smaller particle size of Ni in Ni/C, the ECSA value of NCC was higher than Ni/C due to the lower loading weight than Ni/C (Figure S11, Supporting Information). The LSV curves of each electrocatalyst corrected with the ECSA value were also shown (Figure S12, Supporting Information). To investigate theoretical catalytic properties for the one active site, the Turn Over Frequency (TOF) value was calculated through the methods in the experimental section (Figure 3e). At −0.1 V (vs. RHE), the TOF of NCC was observed to be 3 and 15 times higher than Ni/C and CeO_2_/C, respectively.

To examine the energy barrier of the electrocatalysts, polarization curves were obtained at four different temperatures (Figure S13, Supporting Information). The overpotentials of both NCC and Ni/C decreased with increasing temperatures (34, 39, 44, and 49 °C). To provide a more precise analysis, the linear dependence of ln (j) versus T^−1^ was shown, suggesting that the electrocatalysts exhibited a linear relationship consistent with Arrhenius plots (Figure S14, Supporting Information).^[^ ^24^ ^]^ The Arrhenius activation energy (E_a_) was determined using the Arrhenius equation ln (j) = −E_a_/RT + B.^[^ ^25^ ^]^ The E_a_ values at various overpotentials were plotted in Figure 3f. At an overpotential of 0 mV, the E_a_ values were 28.1 kJ mol^−1^ for NCC and 41.0 kJ mol^−1^ for Ni/C. Additionally, Figure 3g depicts the reaction coordinates of two electrocatalysts. The overpotentials at E_a_ = 0 were determined through extrapolation of Figure 3f. These values were 195.3 mV for NCC and 344.5 mV for Ni/C. The lower activation energy (E_a_) values at η = 0 and at the transition state (E_a_ = 0) for NCC compared to Ni/C were confirmed.

The stability of NCC with Ni/C was compared through the chronopotentiometry (C.P) analysis with −10 mA cm^−2^ (Figure 3h). The degradation rate during 100 h of Ni/C was −900 mV kh^−1^, while NCC had −140 mV kh^−1^, showing ≈6.5 times lower. The overpotential of NCC increased by ≈2 mV, whereas Ni/C experienced an increase of 24 mV (Figure S15, Supporting Information).

When CeO_2_ and Ni existed individually, the two materials showed relatively low performance. Attributed to the EMSI effects, the formation of heterostructures between Ni and CeO_2_ resulted in enhanced electrochemical properties, including an increase in H₂ production and a decrease in activation energy. Furthermore, due to the active electron transfer from the interface between Ni and CeO_2_, the adsorption between the two materials could be further increased, guaranteeing the long‐term stability.

### 2.3 DFT Calculations

**Figure 4:**
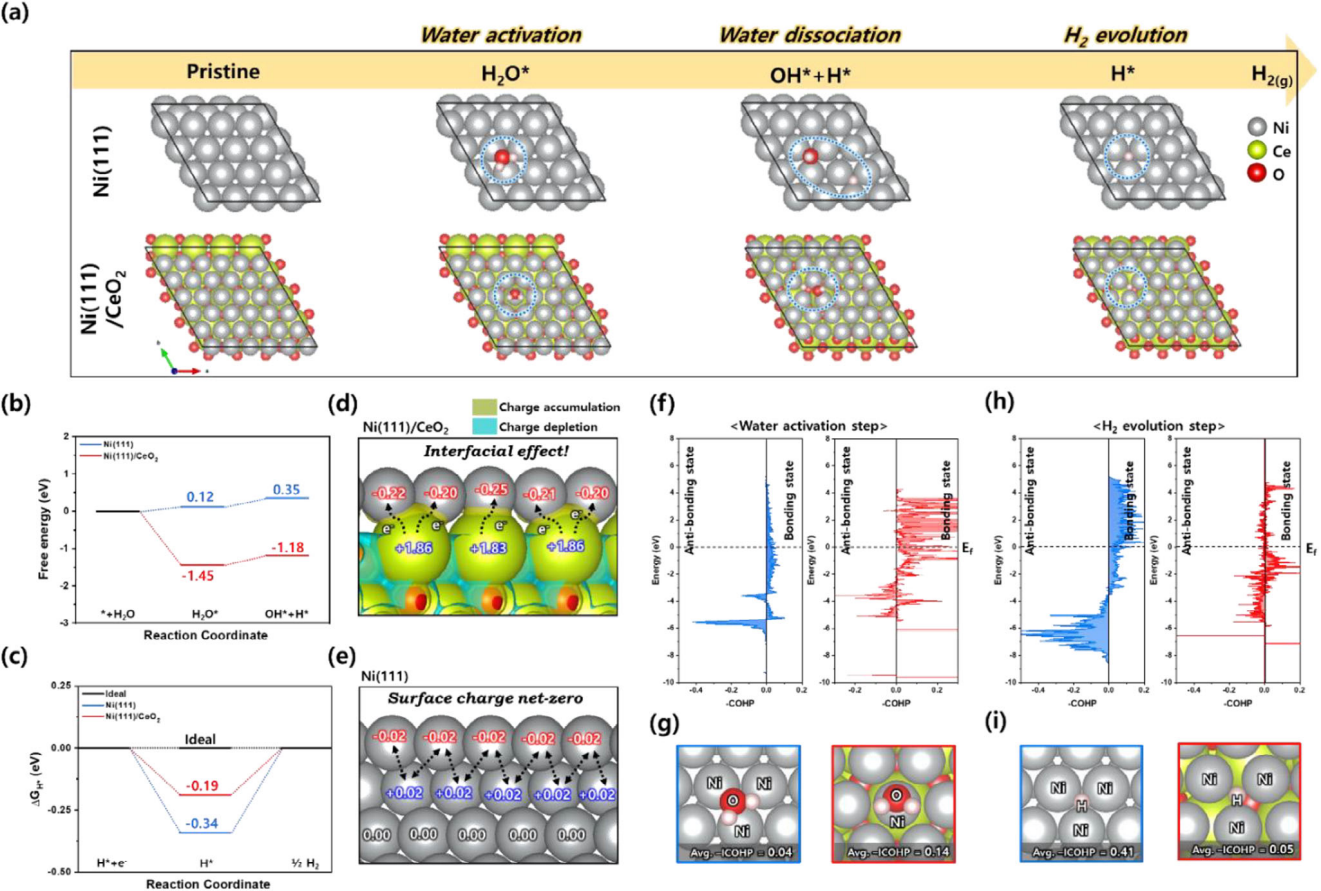
DFT calculations for NCC, Ni/C, and CeO_2_/C. a) The overall reaction steps for alkaline HER include H_2_O adsorption, dissociation, and H_2_ evolution on Ni(111), CeO_2_, and Ni(111)/CeO_2_ (denoted as NCC). The relative energy and the Gibbs free energy for b) water activation and dissociation, and c) Tafel step, respectively, of the designed models. d) The projected density of state (PDOS) and the calculated d‐band center for Ni 3d orbital of Ni(111) and Ni(111)/CeO_2_. e) The charge density differences of Ni(111)/CeO_2_ calculated by the Bader charge analysis. f) –COHP graphs for water activation step and g) H_2_O adsorption model and bonding strength of Ni─O bond from COHP analysis. h) –COHP graphs for H_2_ evolution step and i) H atom adsorption model and bonding strength of Ni─H bond from COHP analysis.

To fundamentally elucidate the synergistic effects at the surface interface of Ni metal and CeO_2_, density functional theory (DFT) calculations were carried out in this study. The catalytic activity is influenced by the electronic structures, which arise from the synergistic interfacial effects between Ni and CeO_2_. First, it is crucial to define a reasonable bulk model for more accurate calculations. The bulk structures of Ni and CeO_2_, which belong to the FM3‐M symmetry group, and the geometrically optimized bulk models are shown in Figure S16 (Supporting Information). Based on these bulk models, the most stable surface structures of Ni(111) and CeO_2_(101) were defined, and an interfacial Ni(111)/CeO_2_(101) model, representing the electrocatalysts in this study, was designed as shown in Figure S17 (Supporting Information). To understand the theoretical catalytic activities toward alkaline HER, the most stable adsorption sites for water adsorption (H_2_O^*^), water dissociation (OH^*^H^*^), and hydrogen atom adsorption (H^*^) were systematically identified on the surfaces of Ni and Ni/CeO_2_ models as summarized in **Figure** **4**a and Figures S18–S20 (Supporting Information) with the following Equation (1):^[^ ^26^ ^]^

(1) $$\Delta E=E_{ads^{*}/surface}-E_{surface}-E_{ads}$$

where Δ*E*, $E_{ads^{*}/surface}$, *E_surface_*, and *E_ads_* were the binding energy, total energy of (H_2_O^*^, OH^*^H^*^, and H^*^ on Ni or CeO_2_), slab energies (Ni and CeO_2_ surface), and isolated energy (H_2_O, OH, O, H), respectively. Moreover, the Gibbs free energy of the reactants and intermediates was calculated as follows in Equation (2):^[^ ^27^ ^]^

(2) $$\Delta G=\Delta E+\Delta E_{ZPE}-T\Delta S$$

where Δ*E* denotes the difference in the DFT calculated ground state energy, Δ*E_ZPE_* denotes the zero‐point energy, which reported values,^[^ ^28^ ^]^ *T* is the temperature at 298 K, and Δ*S* denotes the entropy difference.^[^ ^29^ ^]^

Following adsorption, the water is dissociated into hydroxide anions and adsorbed hydrogen in the Volmer step. Interestingly, Ni/CeO₂ demonstrated spontaneous reaction steps with negative free energies during water activation and dissociation, compared to Ni(111). This suggests that the Volmer step, often considered the bottleneck in overall alkaline HER kinetics, is catalytically alleviated (Figure 4b). Furthermore, hydrogen production follows the Sabatier principle,^[^ ^30^ ^]^ where the ideal Gibbs free energy for adsorbed atomic hydrogen ($\Delta G_{H^{*}}$) should be close to zero. A value closer to zero indicates an ideal catalyst for HER. If $\Delta G_{H^{*}}$ is too large, H^*^ is difficult to adsorb onto the catalyst surface, while if too small, the adsorbed H^*^ struggles to desorb and form H_2_(g). A spontaneous reaction occurs when $\Delta G_{H^{*}}$ is near zero, making it a key descriptor for HER activity. As shown in Figure 4c, the $\Delta G_{H^{*}}$ values for Ni(111) and Ni(111)/CeO_2_ were calculated to be −0.34 and −0.19 eV, respectively. Ni(111)/CeO_2_ exhibited improved catalytic activity for HER, with $\Delta G_{H^{*}}$ being closest to zero. The free energy diagrams predict that Ni(111)/CeO_2_ accelerates the entire process, from water activation to hydrogen production, due to the introduction of interfacial CeO₂, compared to Ni(111). Particularly, from the perspective of kinetics with lower energy barriers, this aligns well with the experimental results in Figure 3g.

To understand the electronic structure modifications between Ni(111) and Ni(111)/CeO_2_, charge density differences and bond lengths at active atoms with adsorbates were investigated using Bader charge analysis and Crystal Orbital Hamilton Population (COHP), respectively. First, the charge density differences between Ni(111) and Ni(111)/CeO_2_ structure were expressed as shown in Equations (3) and (4):^[^ ^31^ ^]^

(3) $$\rho_{1}=\rho_{Ni/Ce_{2}O}-\rho_{Ni}-\rho_{CeO_{2}}$$

(4) $$\rho_{2}=\rho_{Ni}$$

where ρ_1_ and ρ_2_ denote the charge density difference of Ni(111)/CeO_2_ and Ni(111), respectively.

Figure 4d,e, and Figure S21 (Supporting Information) present the charge density differences, where the yellow and cyan areas represent charge accumulation and depletion, respectively. With the introduction of interfacial CeO_2_, the upper‐layered Ni atoms readily acquire negative charges from adjacent Ce atoms, indicating charge transfer from Ce to Ni. This suggests an increase in the charge density of Ni, resulting in a lower oxidation state, which aligns with the negatively shifted binding energies observed in the results of XPS analysis (Figure 2). This phenomenon demonstrates that the modified electronic structure, driven by electron transfer between Ni and CeO_2_, creates a synergistic interfacial effect. This effect accelerates water activation and dissociation while tuning the binding energy to a moderate level, thus enhancing hydrogen production.

Furthermore, to understand and determine the relationship between the bond strength of reactants and intermediates at active sites during the key reaction steps of water activation and hydrogen production, Crystal Orbital Hamilton Population (COHP) analysis was performed. COHP is a computational technique that provides insights into chemical bonding from electronic structures by identifying bonding, non‐bonding, and antibonding interactions between atom pairs. Notably, it can quantify bond strength, with integrated COHP (−ICOHP) serving as a descriptor for bond strength.

For the water activation step (Figure 4f,g), Ni(111)/CeO_2_ exhibited the –COHP values with a higher average Ni─O bond of 0.14, compared to 0.04 for Ni(111). Additionally, the –COHP values with a lower average Ni─H bond for the hydrogen production step were 0.41 for Ni(111) and 0.05 for Ni(111)/CeO_2_, indicating weaker hydrogen adsorption on the interfacial surface of Ni and CeO_2_ due to the synergistic effect (Figure 4h,i). These COHP results from the electronic structure calculations align well with the trends observed in the free energy diagrams in Figure 4b,c.

In addition, the repelling effect of Ni/CeO_2_ against undesired ions, such as Li, Mo, and W, present in the waste alkaline water used in this study (Figure S5, Supporting Information), was confirmed through binding energy calculations. As shown in Figures S22 and S23 (Supporting Information), Ni(111)/CeO_2_ exhibited significantly lower binding energies with these unwanted ions (Li, Mo, W) compared to Ni(111). This suggests that Ni(111)/CeO_2_ can prevent the active sites from being blocked by Li, Mo, and W ions, making it more adaptable electrocatalyst for use in waste alkaline environments through the introduction of an interfacial CeO_2_ layer.

### 2.4 AEMWE Performances

**Figure 5:**
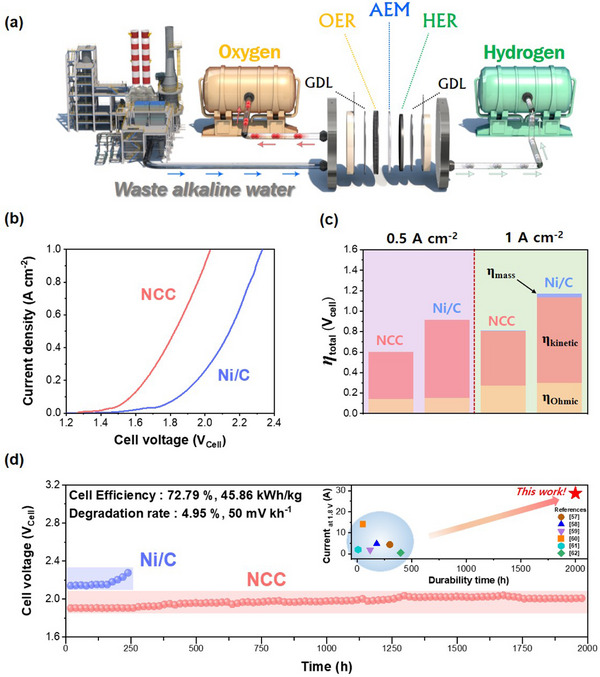
AEMWE performances for NCC and Ni/C. a) The scheme of process scheme for the waste alkaline water electrolysis with AEMWE. b) LSV curves of commercial single cells for NCC and Ni/C. c) The LSV curves were separated into ohmic and kinetic overpotential. d) The long‐term durability analysis for NCC (2000 h) and Ni/C (250 h).

The waste alkaline water from the factory was applied for the practical scale assembly of the AEMWE (**Figure** **5**a). Evaluation of the performance of the AEMWE practical scale cell, encompassing an active surface area of 64 cm^2^, was conducted. Specifically, NCC catalyst was employed at the cathode electrocatalysts, and cobalt oxide (Co_3_O_4_) was used for the anode electrocatalysts.^[^ ^32^ ^]^ Additionally, HQPC‐TMA was utilized for the AEM.^[^ ^33^ ^]^

Before assessing the AEMWE practical‐scale cell, the performance of NCC was evaluated using an AEMWE lab‐scale cell (Figure S24, Supporting Information). The results showing 1.5 A cm^−2^ at 2.0 V demonstrated superior performance than other AEMWE using Ni as cathode electrocatalysts.^[^ ^34^ ^]^ The performance of the AEMWE practical scale cell was assessed through LSV analysis (Figure 5b). The measurement conditions were fixed at waste alkaline water (13.4 pH) and a constant temperature of 60 °C. The performance of the NCC cathode‐based AEMWE exhibited a cell voltage of 1.83 V at 0.5 A cm^−2^ and 2.03 V at 1 A cm^−2^, whereas the Ni/C cathode‐based AEMWE showed 2.14 and 2.33 V at the same current densities. The cell efficiency of NCC was measured at 72.79%, whereas Ni/C exhibited only 61.50%. The energy efficiency for the production of hydrogen by NCC was 45.86 kWh kg^−1^, while Ni/C showed 54.27 kWh kg^−1^.

The influence of interface resistances, such as electrolyte/catalyst and catalyst/substrate, on the overall resistance of the AEMWE practical scale cell was further analyzed utilizing EIS (Figure S25, Supporting Information). The R_s_ serves as a metric for the collective contact resistance at each interface and the electronic and ionic resistance within the components of the AEMWE practical scale cell.^[^ ^35^ ^]^ It was observed that the R_s_ value of the NCC composite was 13% lower compared to that of Ni/C, while the R_ct_ value displayed a decrease of 24% relative to Ni/C. Through overpotential separation, the ohmic, kinetic, and mass overvoltage were investigated (Figure 5c). The ohmic resistance of NCC and Ni/C showed little difference, whereas a significant difference in kinetic resistance between NCC and Ni/C was observed. At 0.5 and 1 A cm^−2^, the kinetic overvoltage of NCC was 0.46 and 0.54 V, respectively, while Ni/C exhibited 0.77 and 0.841 V, respectively. It was demonstrated that NCC catalysts show superior electrocatalytic activity for HER in full‐cell systems compared to Ni/C, consistent with the results obtained in half‐cell analysis.

The long‐term stability analysis for the electrocatalysts NCC and Ni/C was conducted for 2000 and 240 h, respectively (Figure 5d). C.P analysis was carried out at 0.55 A cm⁻^2^ using waste alkaline water at 60 °C. Deterioration of the Ni/C cathode‐based cell began rapidly around 180 h and ceased operation at 240 h. In contrast, the NCC cathode‐based cell maintained a stable performance throughout the 2000 h duration. The NCC cathode‐based cell demonstrated a voltage increment of 50 mV kh^−1^ with a degradation rate of 4.95% over the 2000 h period, while the Ni/C cathode‐based cell exhibited a voltage increment of 643 mV kh^−1^ with a degradation rate of 6.9% over 240 h. Hydrogen production was measured at 17 L h^−1^ under STP (0 °C, 1 atm), and the same amount of hydrogen was confirmed to be measured even after 2000 h. Additionally, the inset Figure showed superior performance and stability of NCC related to the other AEMWE using non‐precious metal both cathode and anode (Table S2, Supporting Information).^[^ ^36^ ^]^

After the long‐term durability analysis of NCC AEMWE, the analysis of the electrocatalysts was performed. The cell efficiency of NCC had a decrement of 3.87% for the 2000 h durability analysis, while Ni/C showed a decrement of 14.17% for the 240‐h durability analysis (Figure S26, Supporting Information). The EIS analysis demonstrated that a small increment of R_s_ occurred, and the semi‐circle for R_ct_ was almost maintained (Figure S27, Supporting Information). The XPS results of the NCC electrode for both before and after durability analysis were shown, concerning the Ni species, the main active site (Figure S28, Supporting Information). Subsequent to the 2000 h durability analysis, Ni^0^ was nearly eliminated, with a significant increase in the proportion of Ni(OH)_2_ at the surface of Ni during the HER. To analyze the structure changes of electrocatalysts, the XRD analysis of the NCC composite was conducted (Figure S29, Supporting Information). After 2000 h durability analysis, the 2‐theta peaks corresponding to Ni and CeO_2_ remained unchanged, while peaks associated with Ni(OH)_2_ were detected.^[^ ^37^ ^]^ The results of XRD and XPS indicated that the surface of Ni underwent transformation to Ni(OH)_2_ during the HER process, including the adsorption and desorption of both hydrogen ions and hydroxyl ions, while the bulk of Ni remained in its metallic state. Furthermore, HR‐TEM and SAED analysis were employed to compare the surface morphology before and after durability analysis (Figure S30, Supporting Information). The overall surface morphology remained largely unchanged during the HER. Additionally, due to the chemical interaction between Ni and CeO_2_, the adsorption of CeO_2_ onto Ni was confirmed. This was further validated through HAADF imaging on a macro scale (Figure S31, Supporting Information). Impurities such as Mo and W were detected on the surface of the NCC after a 2000‐h durability analysis. Further XPS analysis demonstrated the presence of Li, Mo, and W (Figure S32, Supporting Information). Although the TEM EDS and XPS results indicated minimal adsorption of impurities, the NCC demonstrated stable performance and durability in waste alkaline water over a duration of 2000 h. Furthermore, the comparison before and after durability analysis for AEM was also investigated, and little difference in chemical concentrations was confirmed (Figures S33 and S34, Supporting Information).

## 3 Conclusion

The strategy for enhancing the electrochemical properties and durability of Ni‐based HER electrocatalysts was implemented by utilizing the EMSI effect. The electron transfer between Ni and CeO₂ was confirmed through various analytical techniques, leading to improved electrochemical properties due to the modified electronic structure of Ni. DFT calculations were employed to elucidate the underlying mechanisms behind the enhanced catalytic activity and altered charge density distribution, revealing accelerated alkaline HER steps and electronic structure modifications driven by charge transfer from Ce to Ni through the introduction of interfacial CeO_2_ surfaces. The performance of the NCC electrocatalyst was further validated through the assembly of a practical‐scale AEMWE. The NCC catalyst achieved a current density of 1 A cm^−2^ at 2.0 V, in contrast to the Ni/C catalyst, which required 2.33 V at the same current density. Additionally, the hydrogen production efficiency was 72.79% for NCC, compared to 61.50% for Ni/C, and the energy efficiency was demonstrated as 45.86 kWh kg⁻¹ for NCC, compared to 54.27 kWh kg^−1^ for Ni/C. Furthermore, NCC exhibited a degradation rate of only 4.95% with 50 mV kh^−1^ over 2000 h in long‐term durability conducted at 0.55 A cm^−2^, while the Ni/C catalyst showed a degradation rate of 6.9% with 643 mV kh^−1^ over just 240 h.

## Conflict of Interest

The authors declare no conflict of interest.

## Author Contributions

N.I.K, J.L., S.J., and J.J. contributed equally to this work as co‐first authors. N.I.K. and J.L. participated in the experiments and wrote the manuscript. S.J. conducted DFT calculations and wrote the manuscript. J.J. assembled a practical scale of AEMWE and wrote a manuscript. S.W.M. provided Co_3_O_4_ anode electrocatalysts. J.S.H., J.P., and H.L. supported the experiments. M.P., C.K., and J.L. interpreted the results from the experiments. S.K., S.H.Y. provided and analyzed anion exchange membrane. J.Y.L., M.H.S., and S.M.C. supervised and edited the manuscript. All authors discussed the results and co‐wrote the manuscript.

## Supporting information



Supporting Information

## Data Availability

The data that support the findings of this study are available from the corresponding author upon reasonable request.
