# Supplementary material for: Collapsing the Bottleneck by Interfacial Effect of Ni/CeO2 for Long‐Term Hydrogen Production using Waste Alkaline Water in Practical‐Scale Anion Exchange Membrane Water Electrolyzer
Source: Adv Sci (Weinh). 2025 Jun 9;12(34):e02484. doi: 10.1002/advs.202502484 (PMC12442628; doi:10.1002/advs.202502484)
Supplement: Supplementary file 1 — Supporting Information [file ADVS-12-e02484-s001.docx]

**Supplementary information**

Title: Collapsing the Bottleneck by Interfacial Effect of Ni/CeO_2_ for Long-term Hydrogen Production Using Waste Alkaline Water in Practical-scale Anion Exchange Membrane Water Electrolyzer

Nam In Kim, Jaehun Lee, Song Jin, Jaehoon Jeong, Shin-Woo Myeong, Jun Seok Ha, Junyoung Park, Hoseok Lee, Minjeong Park, Chiho Kim, Sungjun Kim, Seok Hwan Yang, Yoo Sei Park, Jooyoung Lee, Jang Yong Lee*, Min Ho Seo* and Sung Mook Choi*

Nam In Kim, Jaehun Lee, Song Jin, Jaehoon Jeong, Shin-Woo Myeong, Jun Seok Ha, Junyoung Park, Hoseok Lee, Minjeong Park, Chiho Kim, Jooyoung Lee and Sung Mook Choi

Energy& Environment Materials Research Division, Korea Institute of Materials Science (KIMS), Changwon 51508, Republic of Korea, E-mail: akyzaky@kims.re.kr

Nam In Kim, Jaehun Lee, Jun Seok Ha

Department of Materials Science and Engineering, Pusan National University, Busan 46241, Republic of Korea.

Song Jin

School of Materials Science and Engineering, Gwangju Institute of Science and Technology (GIST), 261 Cheomdan-gwagiro, Gwangju 500-712, Republic of Korea.

Sungjun Kim, Seok Hwan Yang

Hydrogen Energy Research Center, Korea Research Institute of Chemical Technology, Daejeon, 34114, Republic of Korea.

Yoo Sei Park

Department of Nanoenergy Engineering, Pusan National University, 50, Busandaehak-ro 63 beon-gil 2, Geumjeong-gu, Busan 46241, Republic of Korea

Department of Nano Fusion Technology, Pusan National University, Busandaehak-ro 63 beon-gil 2, Geumjeong-gu, Busan 46241, Republic of Korea

Jang Yong Lee

Department of Chemical Engineering, Konkuk University, Seoul 05029, Republic of Korea. E-mail: lijlij78@konkuk.ac.kr

Min Ho Seo

Department of Nanotechnology Engineering, Pukyong National University, 45 Yongso-ro, Nam-gu, Busan 48547, Republic of Korea. E-mail: forfrit@pknu.ac.kr

Sung Mook Choi

Advanced Materials Engineering, University of Science and Technology (UST), Daejeon 34113, Republic of Korea

**Experimental section**

**1. Synthesis of Electrocatalysts**

**1.1. Preparation of Ni/C and Synthesis of CeO_2_/C**

Ni/C (40 wt%) was purchased from Fuel Cell Store. CeO_2_/C was synthesized by the precipitation method. Cerium (III) nitrate hexahydrate (Ce(NO_3_)_3_·6H_2_O, Aldrich, 99.99%) was used as a precursor for the solution. The carbon black powder (Vulcan XC 72R) (0.6 g) was dispersed in Ethanol (100 ml). The Cerium precursor (1.2 g) was added to the carbon-suspended solution and mixed with vigorous stirring. The pH of the mixed solution was adjusted to 10 by adding drops of NH_4_OH solution. Then the solution was stirred for 1 h and was washed with ethanol and vacuum filtration. The obtained cake was lyophilized at -70 ℃ for 24 h. The dried powder was annealed at 400 ℃ for 4 h in a N_2_ atmosphere.

**1.2. Synthesis of Ni-CeO_2_/C (NCC) and Preparation of Ni/C**

Ni/CeO_2_/C (NCC) was synthesized by the co-precipitation method. Nickel (II) nitrate hexahydrate (Ni(NO_3_)_2_·6H_2_O, Aldrich, 99.99%) and Cerium (III) nitrate hexahydrate were used as precursors for the solution. The carbon black powder (0.4 g) was dispersed in Ethanol (250 ml). The Nickel precursor (2.0 g) and the Cerium precursor (0.6 g) were added to the carbon-suspended solution and mixed with vigorous stirring. The pH of the mixed solution was adjusted to 10 by adding drops of NH4OH solution. Then the solution was stirred for 1 h and was washed with ethanol and vacuum filtration. The obtained cake was lyophilized at -70 ℃ for 24 h. The dried powder was annealed at 240 ℃ for 2 h, and then increased to 500 ℃ and annealed for 4 h in 200 SCCM of H_2_ (4%) + N_2_ (96%) atmosphere.

**2. Materials Characterization**

Thermo Fisher Scientific, Talos F200X was employed for Transmission Electron Microscopy (TEM) and Electron Energy Loss Spectroscopy (EELS) analysis. X-Ray Diffraction (XRD) analysis was conducted with Malvern PANalytical Empyrean Series 3 X-ray diffractometer equipped with Cu-Kα radiation (40kV, 30mA) as the photon source. X-ray Photoelectron Spectroscopy (XPS) analysis was performed by NEXSA, Thermo Fisher Scientific. Inductively Coupled Plasma – Optical Emission Spectrometer (ICP-OES) analysis was conducted with PerkinElmer (Avio500&Avio550) and Ion Chromatograph (IC) analysis was investigated through Metrohm (940 Professional IC Vario).

**3. Electrochemical investigation**

**3.1. Half-Cell Analysis**

The VMP-3 (BioLogic) 3-electrode system was utilized for evaluating the electrochemical performances of each electrocatalysts. The reference electrode utilized was a Hg/HgO electrode, while a carbon rod served as the counter electrode. PTFE (Poly Tetra Fluoro Ethylene) platinum electrode holder was used for the working electrode. The Catalysts-Coated Substrate (CCS) method was used. Each electrocatalysts were coated on the microporous layer carbon cloth stuck by Nafion. The loading weights of Ni in coated NCC was approximately 4 mg cm^-2^ and Ni in coated Ni/C (FC catalysts 40 wt% on Vulcan) was about 5 mg. The waste alkaline solution (pH 13.4) was maintained during experimentation. Linear Sweep Voltammetry (LSV) scans were conducted at a scan rate of 1 mV sec^-1^, ranging from -0.85 to -1.6 V (vs. Hg/HgO), with 85% IR-correction, which can avoid excessive correction.

**3.2. Calculation about theoretical value for volume of H_2_ and O_2_**

The theoretical value of H_2_ and O_2_ production was calculated through the Eq.1.

| $H_{2} Volume \left( \frac{ml}{min} \right)=Current density \left( \frac{mA}{{cm}^{2}} \right)\left( \frac{1}{1.6*{10}^{-19} C} \right)\left( \frac{1}{6.02*{10}^{23}} \right)\left( \frac{1}{2} \right)\left( 22.4 L \right)*(\frac{60sec}{min})$ | Eq.1 |
| --- | --- |

The theoretical volume of O_2_ was calculated to be twice the volume of H_2_, reflecting that the OER involves two times more electrons than the HER.

**3.3. Calculations about ECSA and TOF**

The ECSA (m^2^) was calculated through the eq.2.

|  | $ECSA=\frac{C_{dl}}{C_{s}}$ | Eq.2 |
| --- | --- | --- |

The capacitance of the double layer (C_dl_) was calculated with different scan rates (1 mV sec^-1^ ~10 mV sec^-1^) and the value of R squared was over 0.99. For specific capacitance (C_s_), in alkaline solution, 0.04 mF cm^-2^ was used.[1] Ce in CeO_2_/C and Ni in NCC and Ni/C was used for metal loading weights.

Turnover frequency (TOF) was calculated under the assumption of 100% Faradaic efficiency through the Eq.3.

|  | $TOF=\frac{The number of Hydrogen turnover (s^{-1})}{The number of active sites (sites)}$ | Eq.3 |
| --- | --- | --- |

The number of hydrogen turnover were calculated with current density (mA cm^-2^), Faraday constant (96485 C mol^-1^) and the number of Avogadro (6.022 *10^23^) through the Eq.4.

| ${\#}_{H_{2} Turnover} (s^{-1})=\left( j \frac{mA}{{cm}^{2}} \right) \left( 1 {cm}^{2} \right)\left( \frac{A}{1000 mA} \right)\left( \frac{1}{96485}\frac{mol}{C} \right)\left( 6.02*{10}^{23} \frac{numbers}{mol} \right)\left( \frac{1 mol}{2 mol of e^{-}} \right)$ | Eq.4 |
| --- | --- |

The number of active sites was calculated with the ECSA (m^2^ g^-1^), loading weights (μg cm^-2^), charge density (μC cm^-2^), Faraday constant (96485 C mol^-1^) and the number of Avogadro (6.022 * 10^23^) through the Eq.5.

| ${\#}_{Active sites}(sites)=\left( ECSA \frac{m^{2}}{g} \right) \left( 1 {cm}^{2} \right)\left( {weights}_{loading}\frac{g}{m^{2}} \right)\left( Charge density \frac{C}{{cm}^{2}} \right)\left( \frac{1}{96485}\frac{mol}{C} \right)\left( 6.02*{10}^{23} \frac{sites}{mol} \right)$ | Eq.5 |
| --- | --- |

**3.4. Activation Energy**

The activation energy (E_a_) of each electrocatalyst was calculated through the Arrhenius equation. LSV curves with four different temperatures were investigated. The current densities (j) at different potentials were transformed to ln j. The slope from the linear relationship between ln j and T^-1^ included the E_a_ and gas constant (R).

**4. Computational details**

To elucidate the electronic interfacial effects between Ni and CeO₂, first-principles density functional theory (DFT) calculations using the Vienna Ab initio Simulation Package (VASP) were employed in this study.^[2]^ The projector augmented wave (PAW) method was used to efficiently simulate core electron interactions for the electronic structure calculations.[3] To account for electron exchange and correlation, the generalized gradient approximation (GGA) was employed, with the Revised Perdew-Burke-Ernzerhof (RPBE) functional applied to mitigate the overestimation of binding energies with adsorbates in the designed models.[4, 5] The energy and force convergence criteria for the self-consistent field calculations were set to be smaller than 0.05 eV Å⁻¹ and 10⁻⁴ eV, respectively, for maximum atomic forces and complete ionic relaxation. A cut-off energy of 520 eV was used, and the Methfessel-Paxton smearing method was applied for improved ionic and geometric optimization,[6] with a 30 Å vacuum gap along the z-direction to prevent interactions between structures within the unit cell. Additionally, a Monkhorst-Pack k-point mesh of (6 × 6 × 1) was used for total energy calculations and structural optimizations. Bader charge analysis, incorporating Blöchl corrections based on the tetrahedron method,[7] was conducted to calculate the charge density differences for the Ni(111) and Ni(111)/CeO₂ models. Crystal Orbital Hamilton Population (COHP) calculations were also performed to explore the bond lengths between adsorbates and active sites on the prepared surfaces.[8, 9]

**5. AEMWE assembly**

**5.1. lab scale AEMWE**

The lab scale AEMWE was assembled with MEA using HQPC-TMA (70 μm) for the membrane, NCC for a cathode electrocatalyst and Co_3_O_4_ for an anode electrocatalyst. The polarization curve was investigated by the VMP3B-20 (Biologic). The scan rate was 1 mV sec^-1^ and the flow rate was 100 ml min^-1^. The Nickel foam and Carbon cloth were used as PTL for the anode and cathode, respectively. The surface area of the anode and cathode were 7.1 cm^2^ and 4.9 cm^2^, respectively.

**5.2. Practical scale AEMWE**

The HQPC-TMA (KRICT, Daejeon, Republic of Korea) anion exchange membrane of 70-micrometer thickness, with an area of 79 cm^2^, was used to exchange the anions and separate the hydrogen and oxygen. The membrane was immersed in 1.0 M KOH and soaked for 24 h before use due to ion exchange. At the anode with an area of 79 cm^2^, nickel foam substrate was used for the porous transport layer (gas/liquid), and cobalt oxide catalyst was used for the anode. At the cathode with an area of 64 cm^2^, a microporous layered carbon cloth was used for the gas diffusion layer. The NCC and Ni/C catalysts were used for the cathode prior to measuring the electrochemical activity and long-term durability of the AEM water electrolyzers, the temperature of the electrolyte increased at a rate of 1 ^o^C per minute to ensure sufficient heating and hydrophilic wetting of the integrated MEA, and it was circulated at a flow rate of 1.2 L min^-1^ for approximately 2 h. After a sufficient warm-up of the cell, the characteristic evaluation was performed. All measurement conditions were as follows. The AEMWE was conducted under controlled conditions, with waste-alkaline water at a pH of 13.4, a temperature of 60°C, and an electrolyte circulation flow rate of 1.2 L/min. The laboratory conditions were maintained at a room temperature of 25°C and a humidity level of 35%. The performance of the AEMWE large-area single 1-cell was assessed through LSV analysis using HCP-803 (Biologic) potentiostat. The voltage was scanned from 1.2 V to 2.0 V at a scan rate of 1 mV s^-1^ to evaluate the polarization curves and the current density was normalized to the active surface area of 64 cm^-2^. Galvanostatic electrochemical impedance spectroscopy (GEIS) measurements were carried out at a constant current density of 0.55 A cm^-2^ and a frequency range from 10 kHz to 100 mHz, using the HCP-803 (Biologic) potentiostat. The long-term stability of the AEM water electrolyzer single 1-cell was evaluated under constant current density operation to assess the degradation behavior and durability. The analysis was conducted at a constant current density of 0.55 A cm^-2^ for 2,000 h. The amount of generated hydrogen was measured in two ways: (1) using a bubble meter and (2) using a mass flow meter installed in the evaluation equipment. The amount of generated hydrogen was calculated by measuring for 60 seconds under a current density condition of 0.55 A cm^-2^.


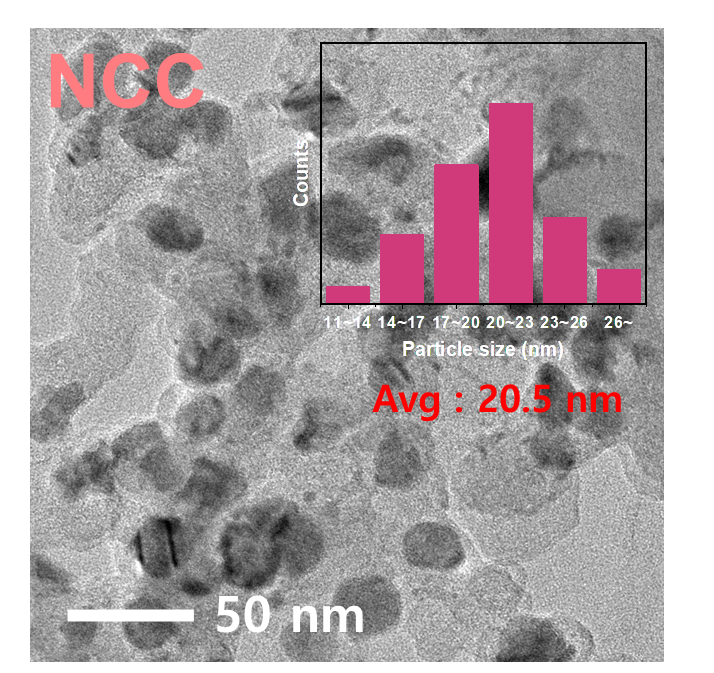


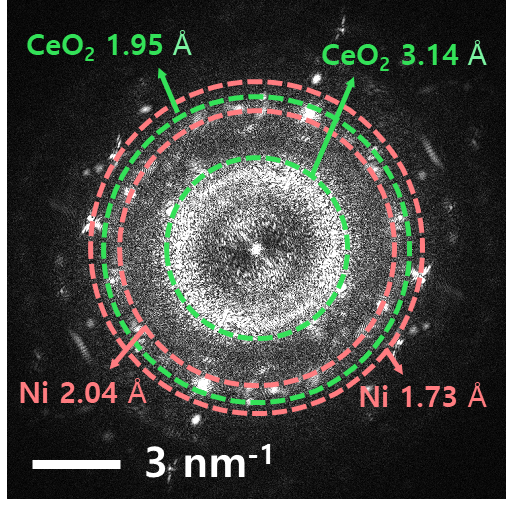


**Figure S1** HR-TEM image of NCC. Inset: average particle size of Ni.

**Figure S2** SAED pattern of NCC calculated through FFT.


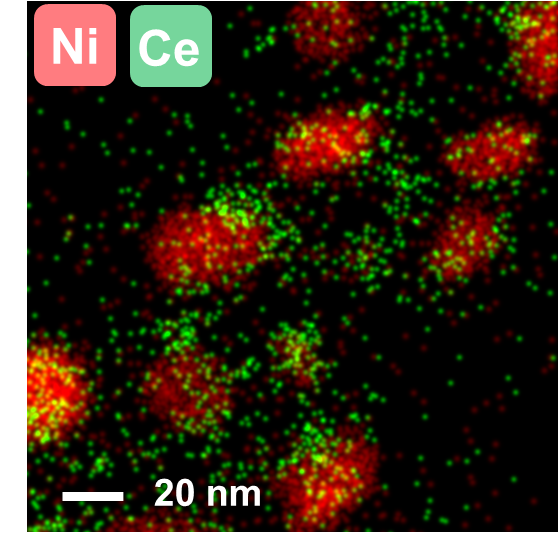


**Figure S3** HAADF image of macro scale for NCC.


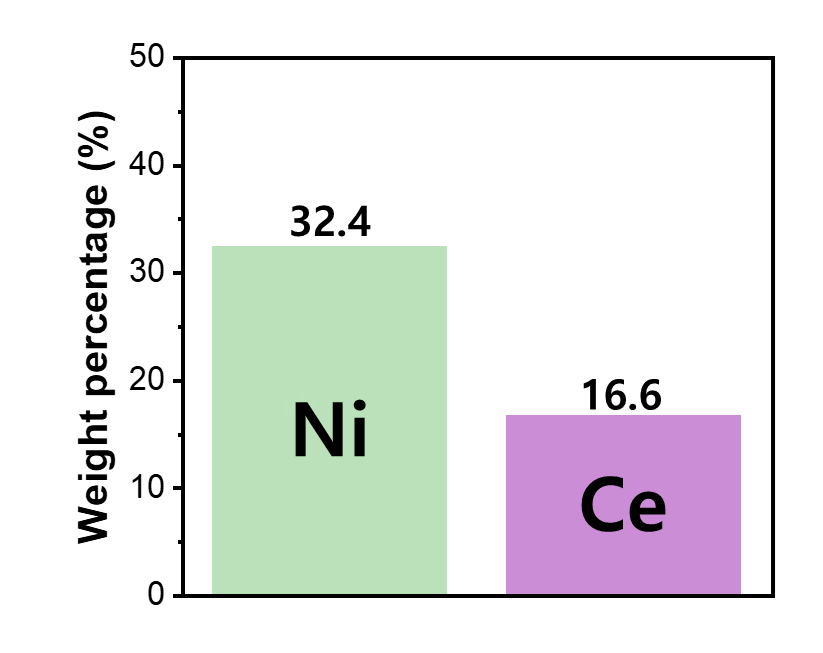


**Figure S4** ICP-OES results of NCC for investigation of Ni and Ce concentration.


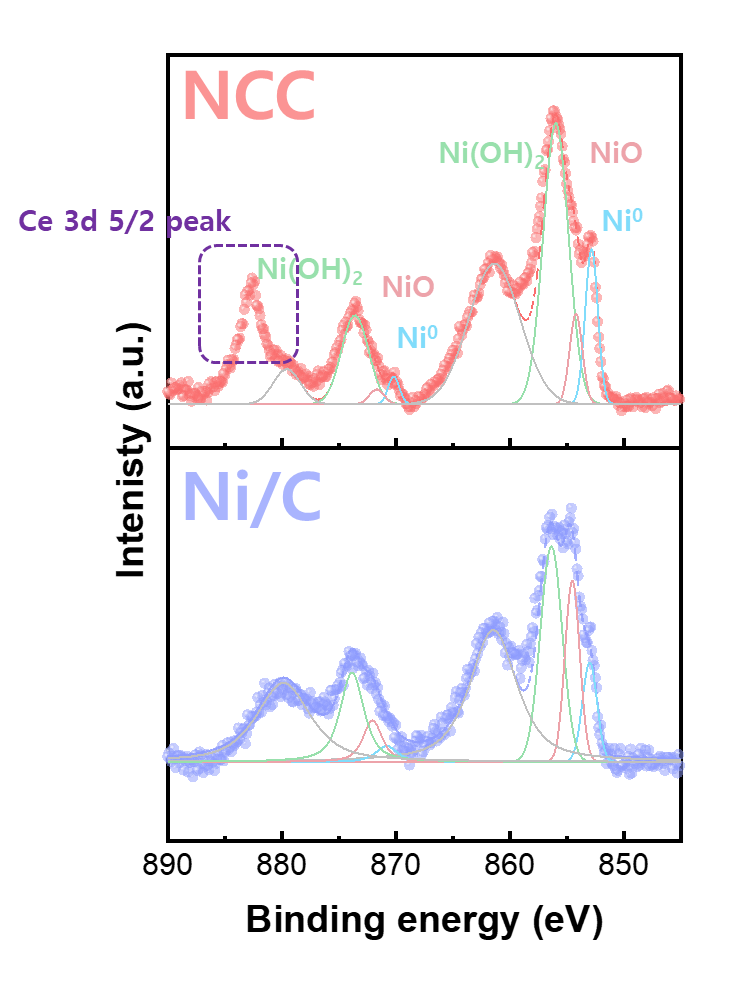


Figure S5 XPS results of Ni 2p peak for NCC and Ni/C


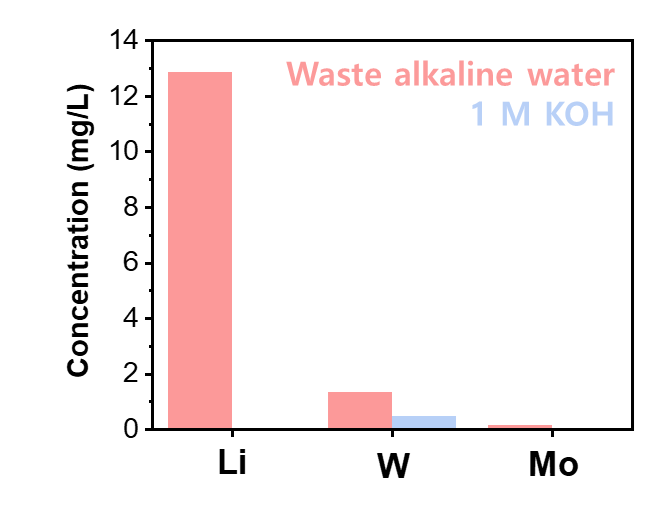


**Figure S6** Ionic concentration plot of Waste alkali and 1 M KOH electrolyte.


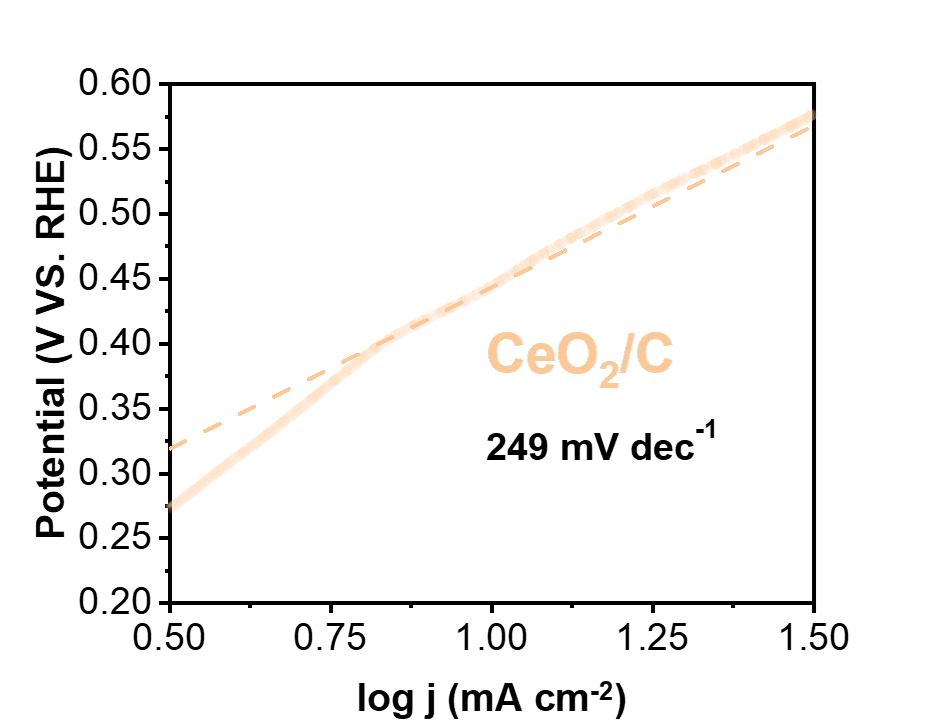


**Figure S7** Tafel slope of CeO_2_/C.


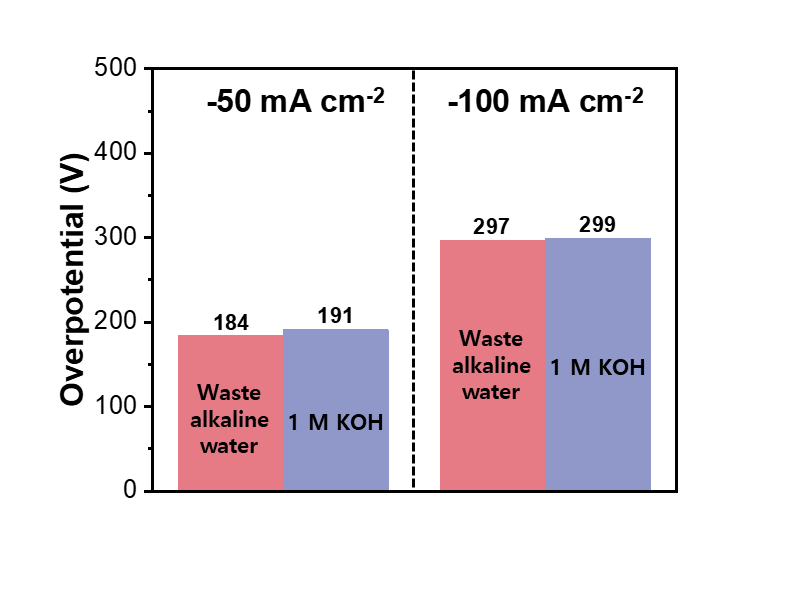


**Figure S8** The overpotential of NCC in waste alkaline water and 1 M KOH electrolyte at current density of -50 and -100 mA cm^-2^, respectively.


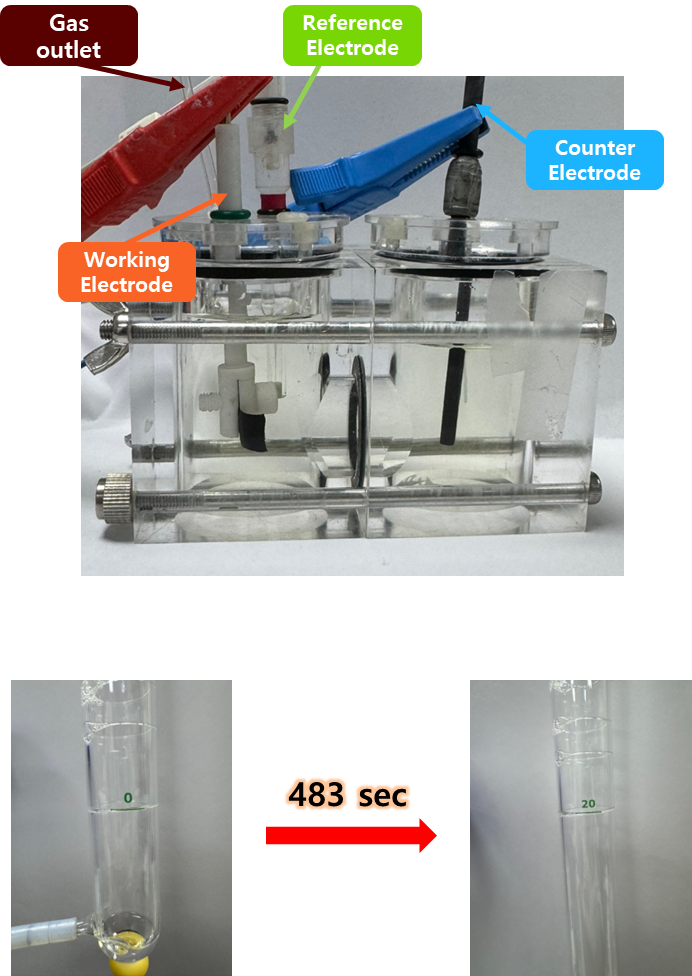


**Figure S9** H-type cell with 3-electrodes system in waste alkaline water media.


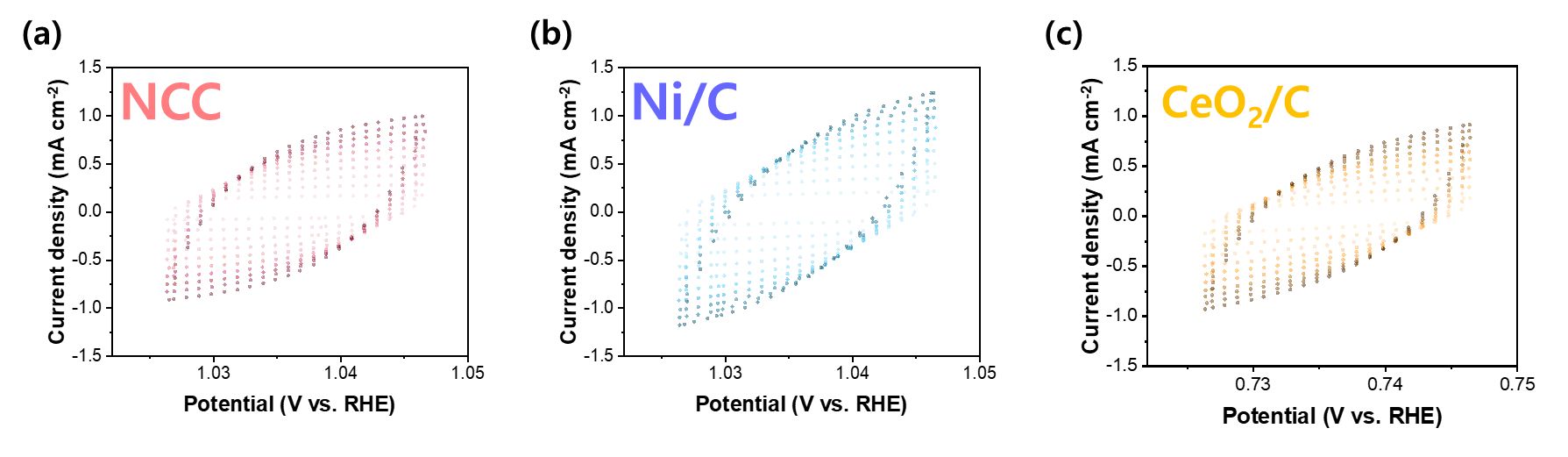


**Figure S10** CV curves with different scan rate (1 ~ 10 mV sec^-1^) of **(a)** NCC, **(b)** Ni/C and **(c)** CeO_2_/C.


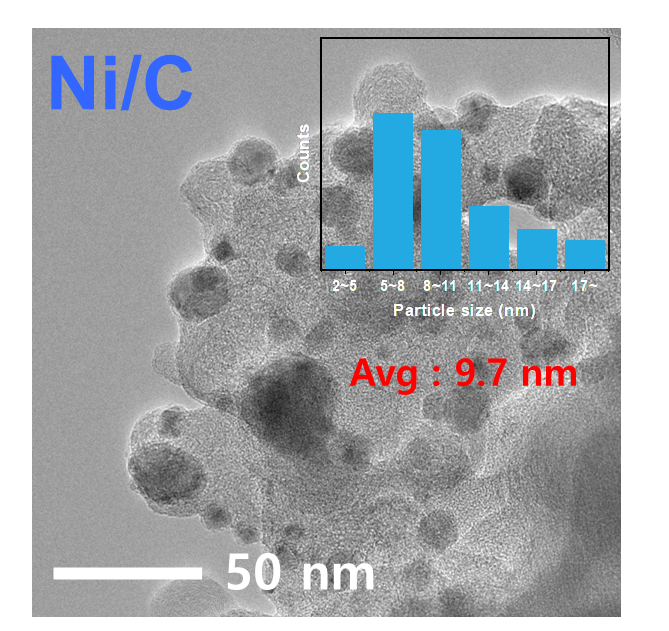


**Figure S11** HR-TEM image of Ni/C. Inset: average particle size of Ni.


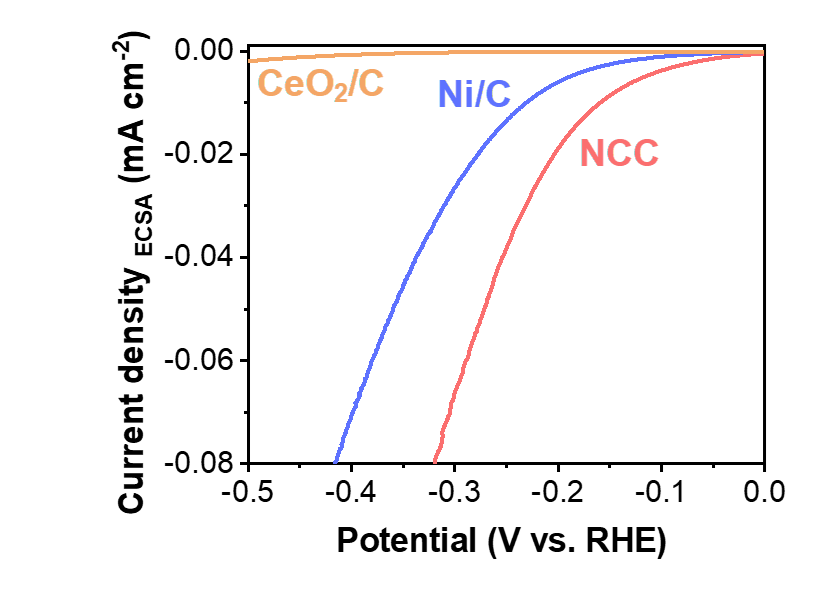


**Figure S12** ECSA normalized HER performance of NCC, Ni/C and CeO_2_/C.


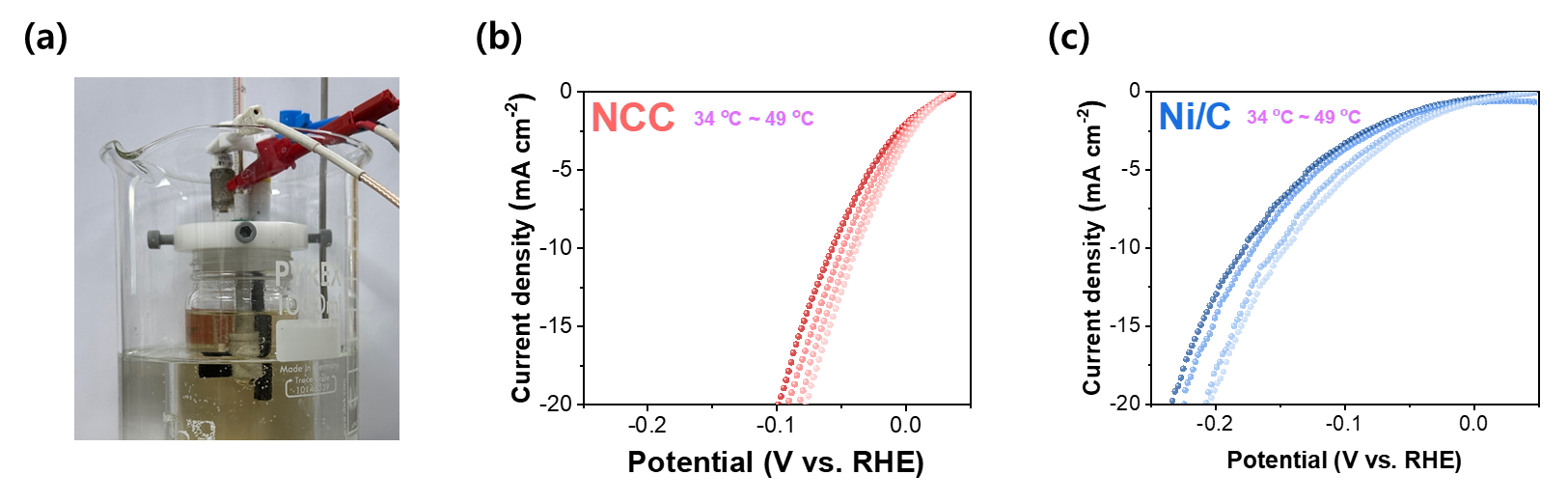


**Figure S13 (a)** The photograph of 3-electrode cell for measuring activation energy. The LSV curves of **(b)** NCC and **(c)** Ni/C with different temperature (34, 39, 44, 49 ℃).


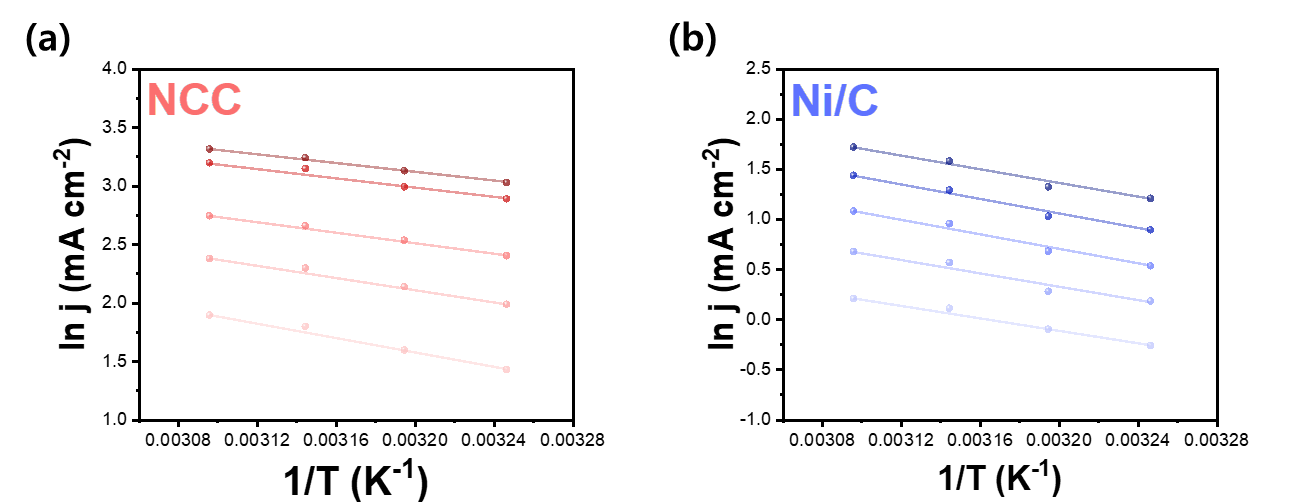


**Figure S14** The relation of temperature with current for **(a)** NCC and **(b)** Ni/C were calculated through the Arrhenius equation.


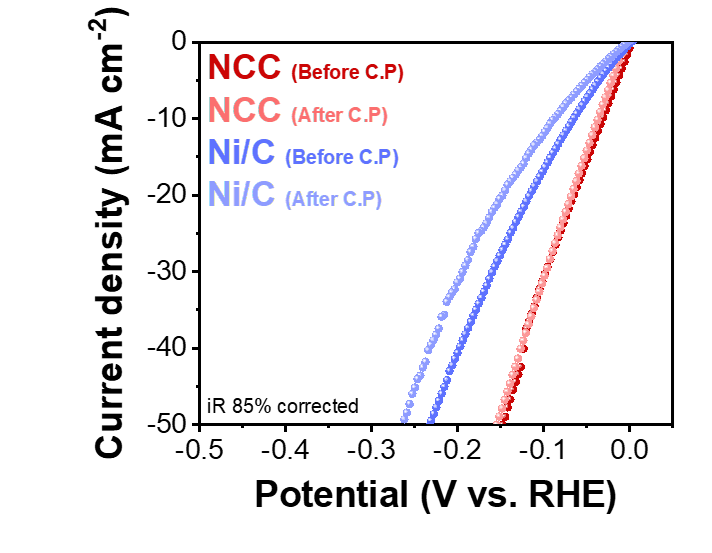


**Figure S15** HER performance of NCC and Ni/C before and after the C.P analysis.


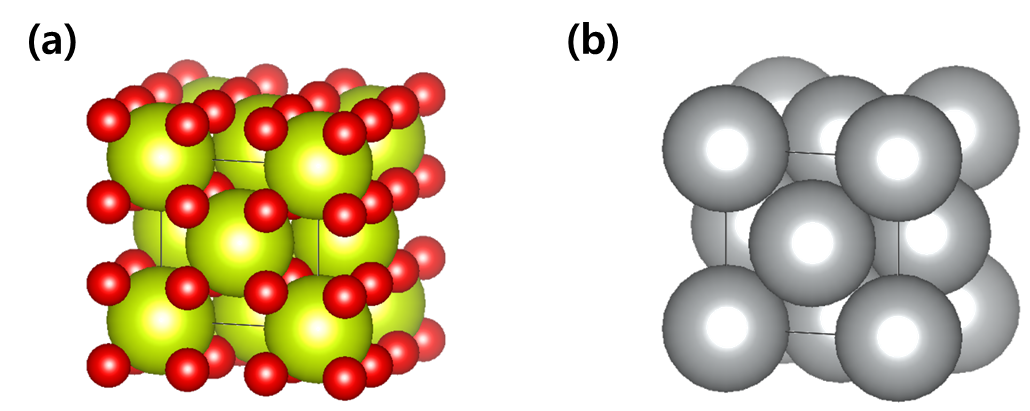


**Figure S16** The bulk model structures of **(a)** CeO_2_ and **(b)** Ni.


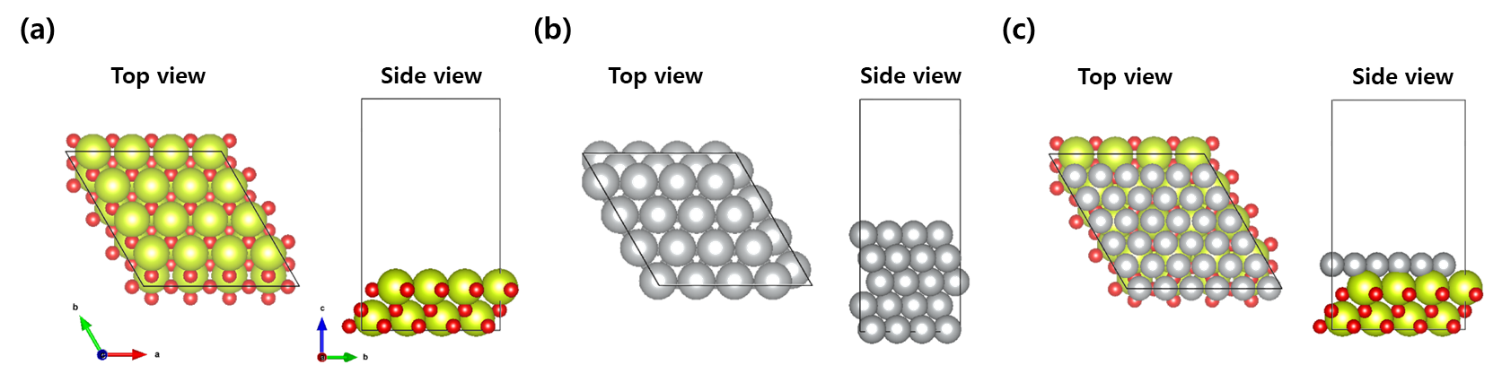


**Figure S17** The optimized surface model structures of **(a)** CeO_2,_ **(b)** Ni and **(c)** Ni(111)/CeO_2_ on the top and side view.


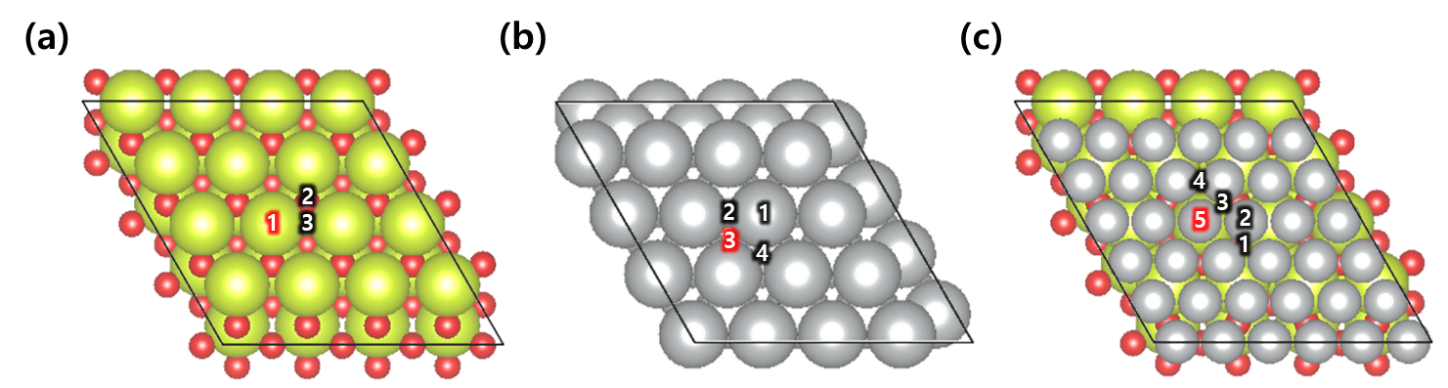


**Figure S18** Model structures considered the possible adsorption sites of H_2_O* on **(a)** CeO_2_, **(b)** Ni and **(c)** Ni(111)/CeO_2_ to find out the most stable site (highlighted as red color).


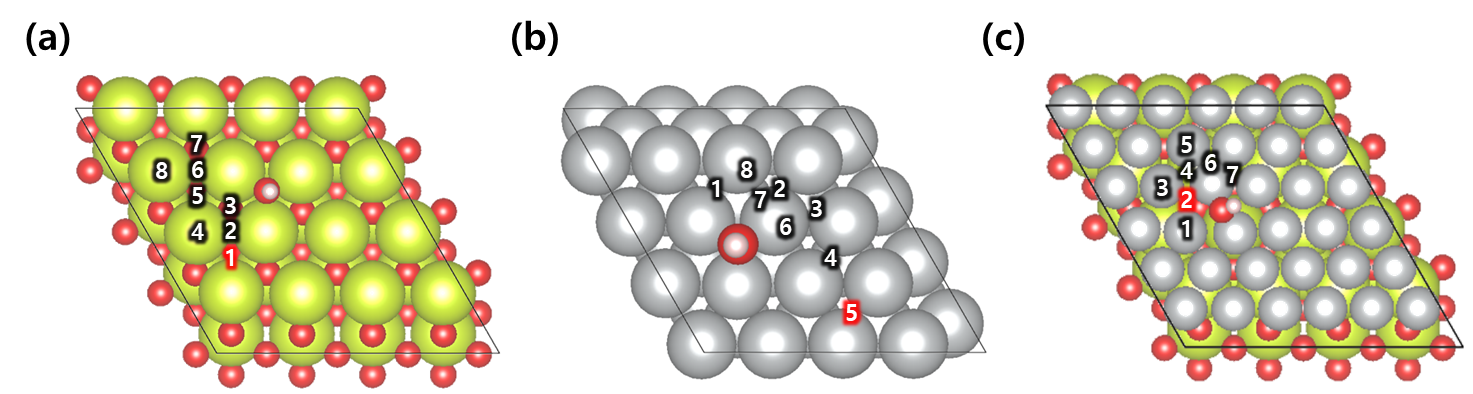


**Figure S19** Model structures considered the possible dissociation sites from H_2_O* to OH*H* on **(a)** CeO_2_, **(b)** Ni and **(c)** Ni(111)/CeO_2_ to find out the most stable site (highlighted as red color).


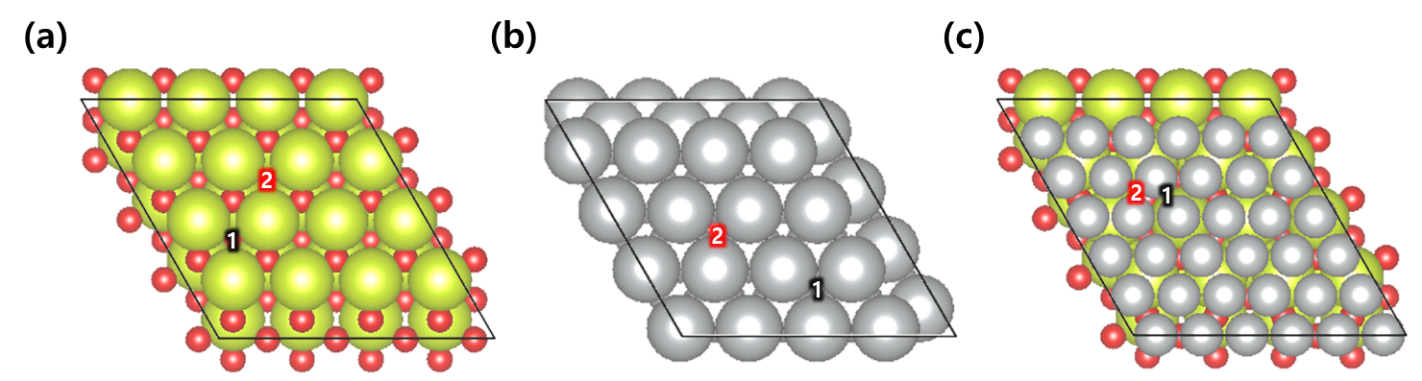

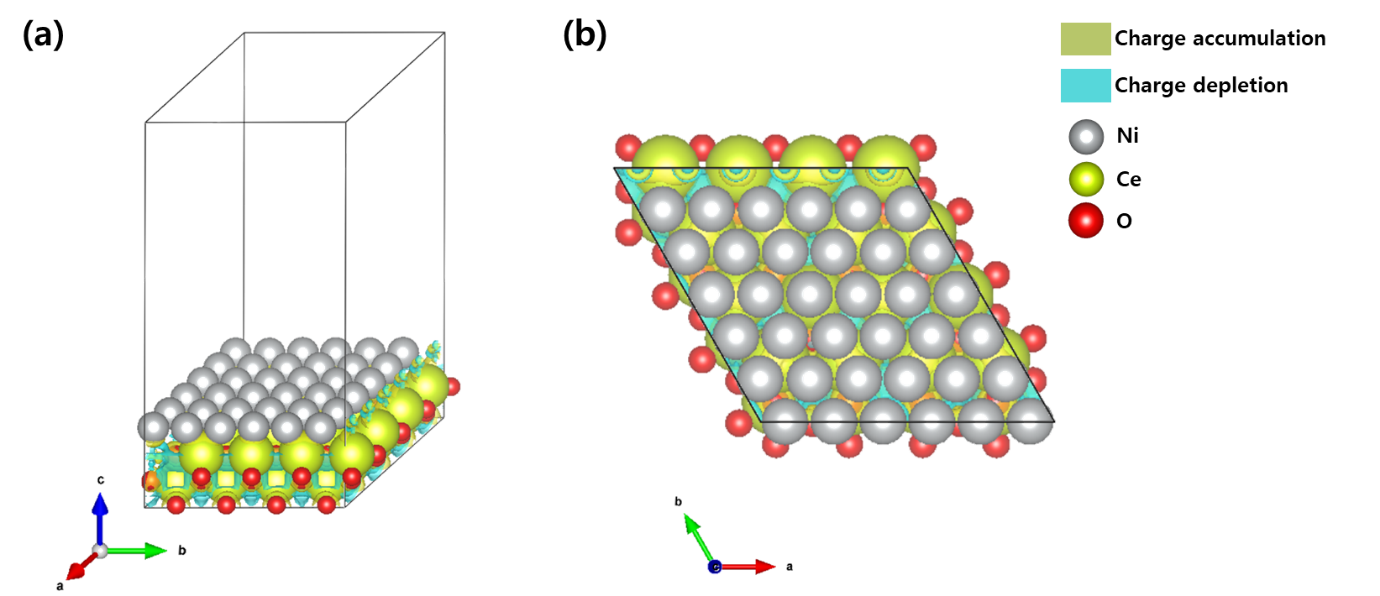


**Figure S20** Model structures considered the possible adsorption sites of H* on **(a)** CeO_2_, **(b)** Ni and **(c)** Ni(111)/CeO_2_ to find out the most stable site (highlighted as red color).

**Figure S21** Charge density differences of Ni(111)/CeO_2_ on (a) side view (b) and top view.


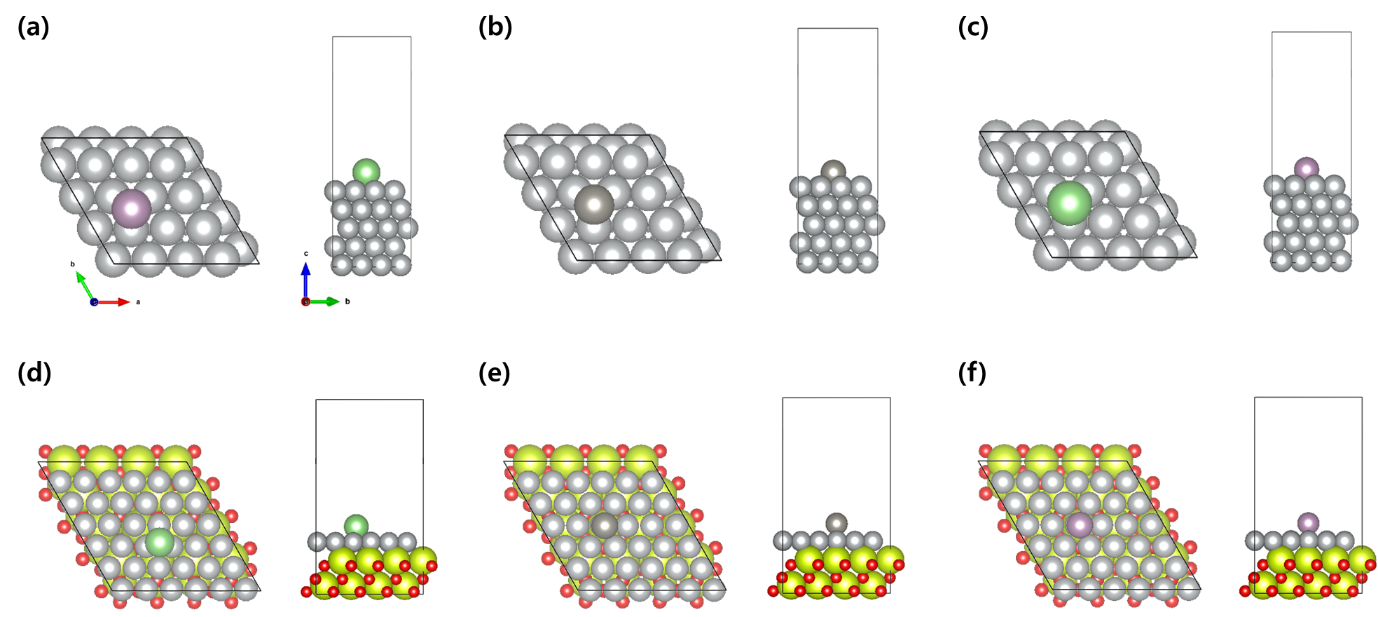


**Figure S22** Model structures of the unexpected ions (Li, W, Mo) adsorbed on (a-c) Ni(111) and (d-f) NCC.


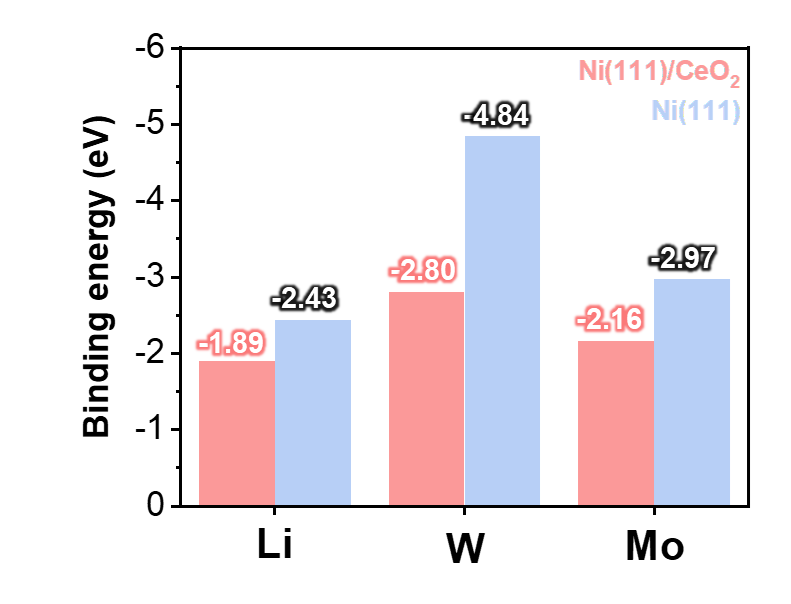


**Figure S23** The comparison for binding energies of the unnecessary ions (Li, W, Mo) on Ni(111) and NCC.

**Figure S24** AEMWE single cell performance of NCC in Waste alkaline electrolyte at 60 ℃.


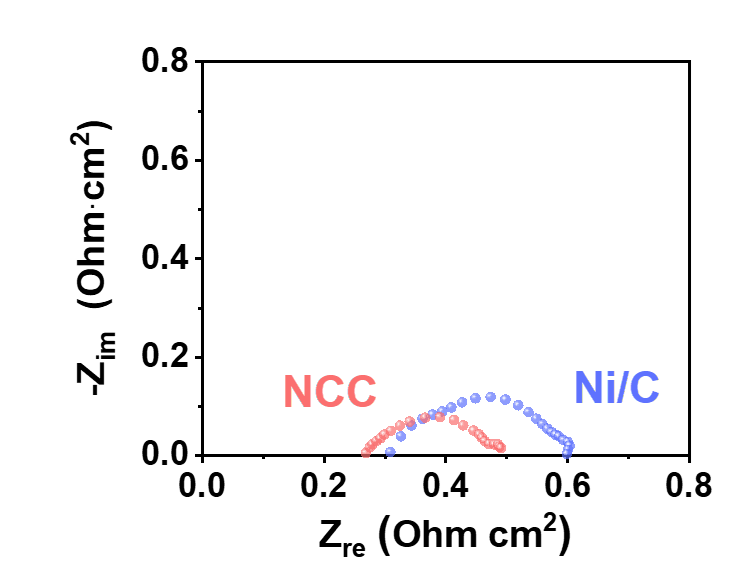


**Figure S25** Impedance of NCC and Ni/C investigated at AEMWE


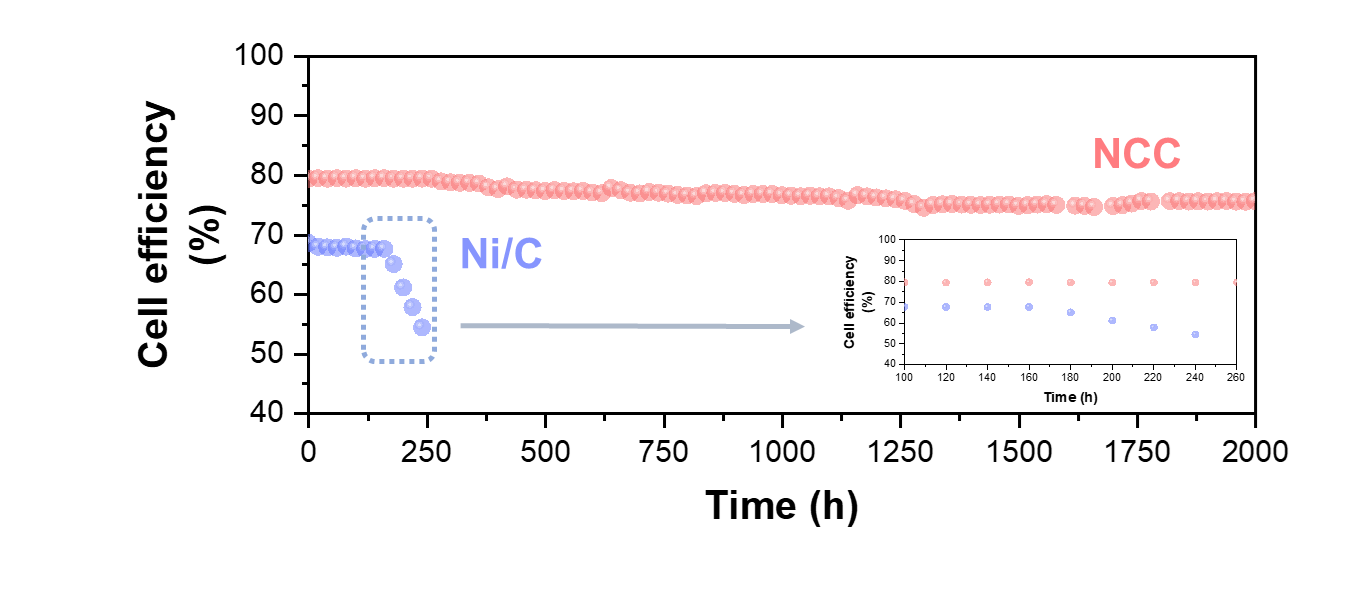


**Figure S26** Cell efficiency plots of NCC and Ni/C during durability analysis. Inset: zoomed-in graph.


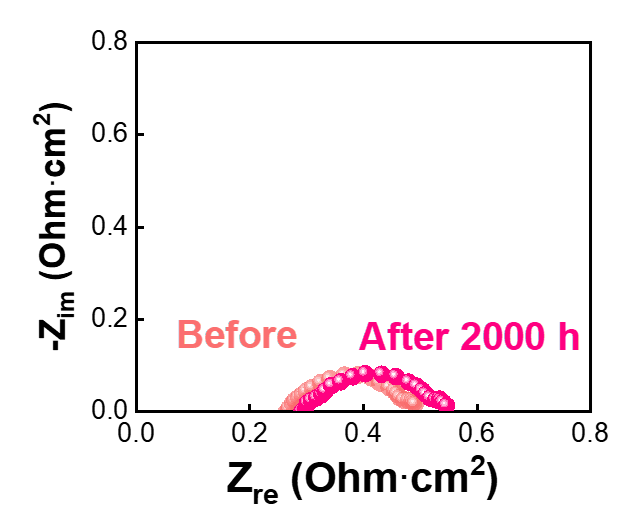


**Figure S27** EIS plots of practical scale AEMWE before and after durability analysis.


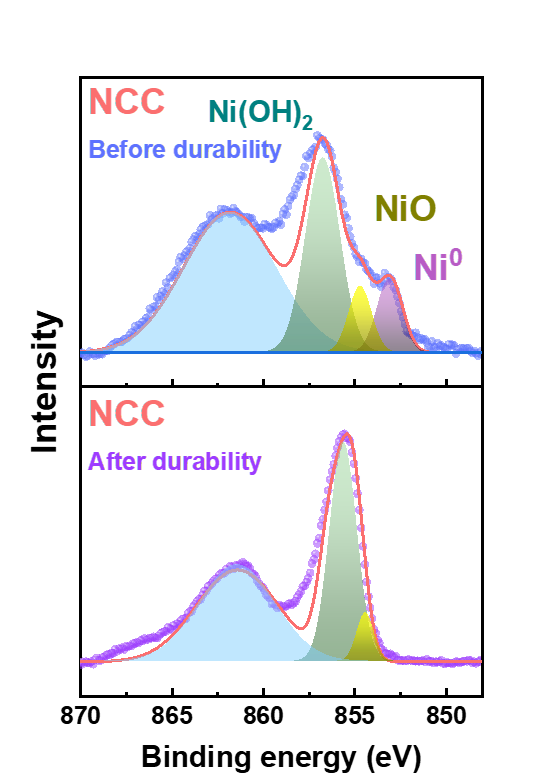

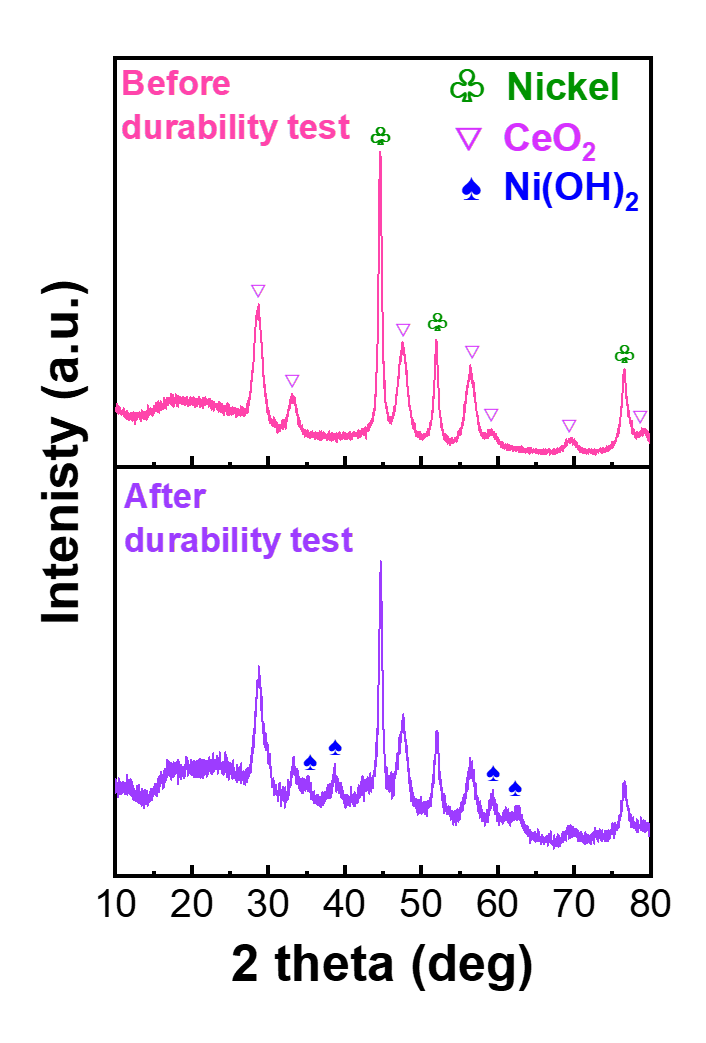


**Figure S28** XPS analysis of NCC for before and after durability analysis

**Figure S29** XRD investigation of NCC for before and after durability analysis


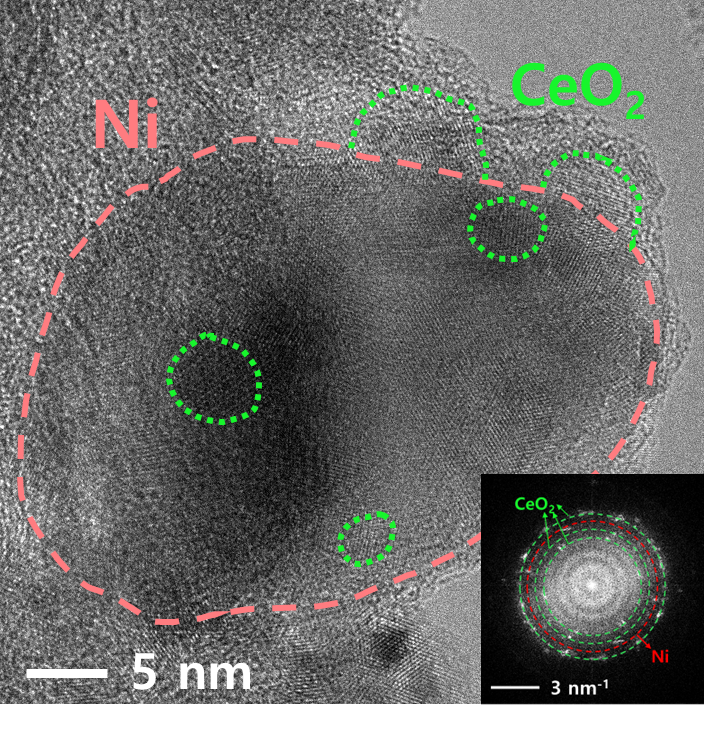


**Figure S30** HR-TEM image and SAED pattern of NCC after 2000 h durability analysis.


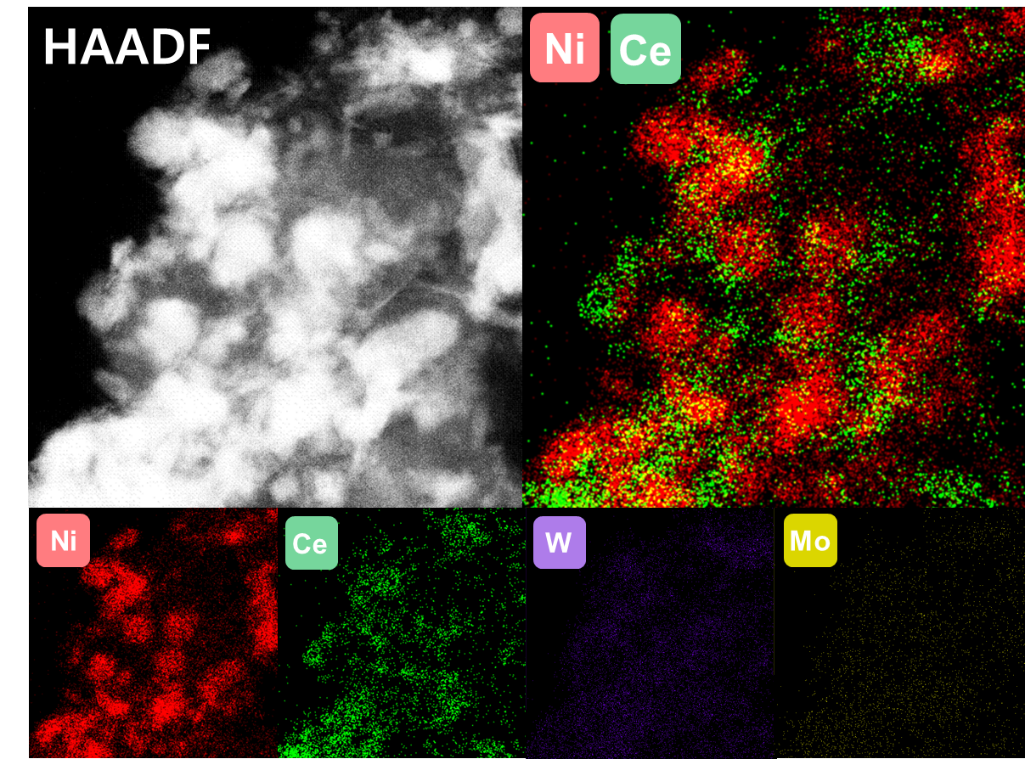

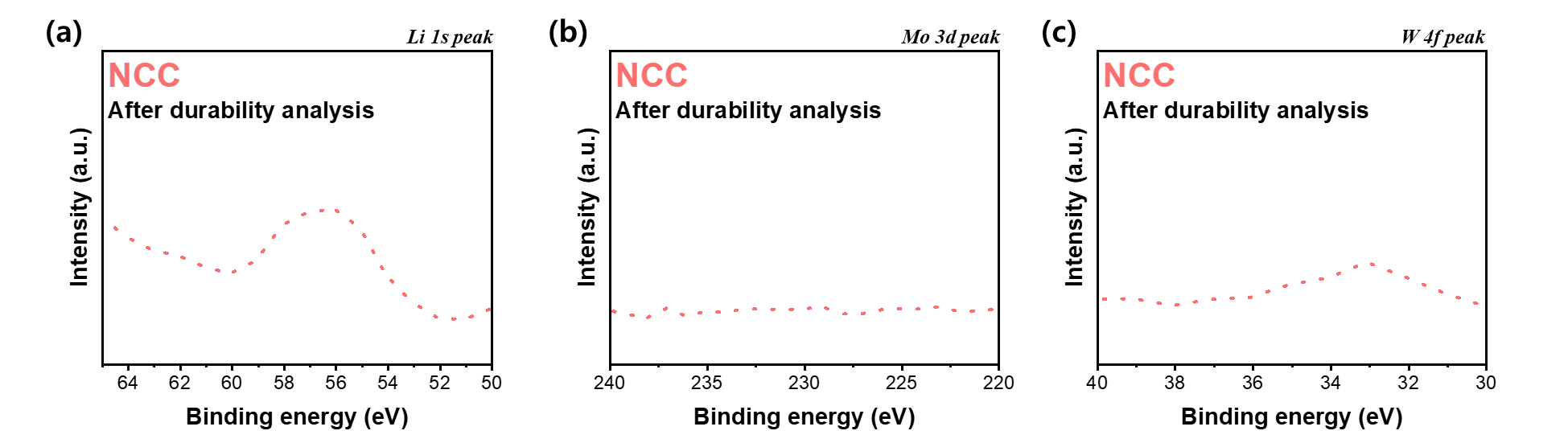


**Figure S31** HAADF- EDS mapping image of NCC after 2000 h durability analysis.

Figure S32 XPS results of NCC for post durability analysis for each impurities. (a) Li, (b) Mo and (c) W.


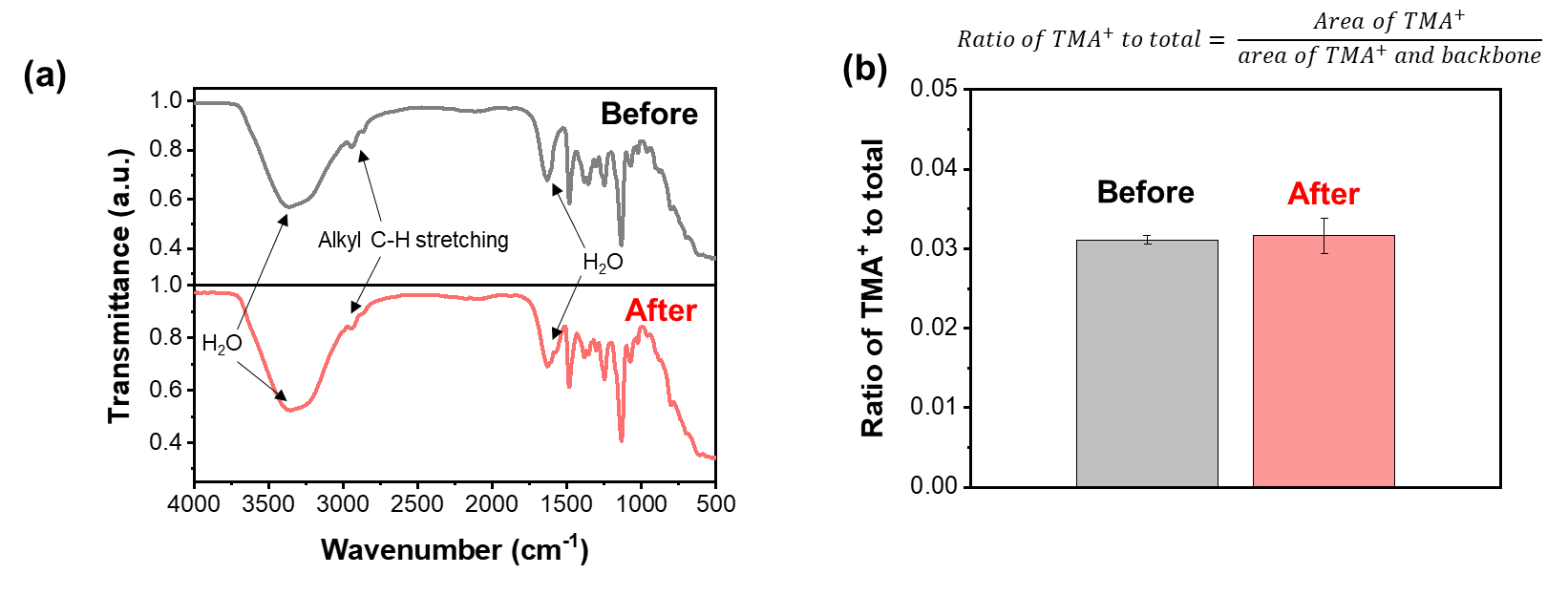


**Figure S33** Structural analysis of HQPC-TMA membranes, before and after the durability analysis. **(a)** FT-IR spectra of HQPC-TMA membranes. **(b)** The ratio of observed TMA^+^ peak to the total polymer peaks (area of TMA^+^ peak / area of TMA^+^ and backbone peaks) calculated from positive ToF-SIMS spectra of membranes.


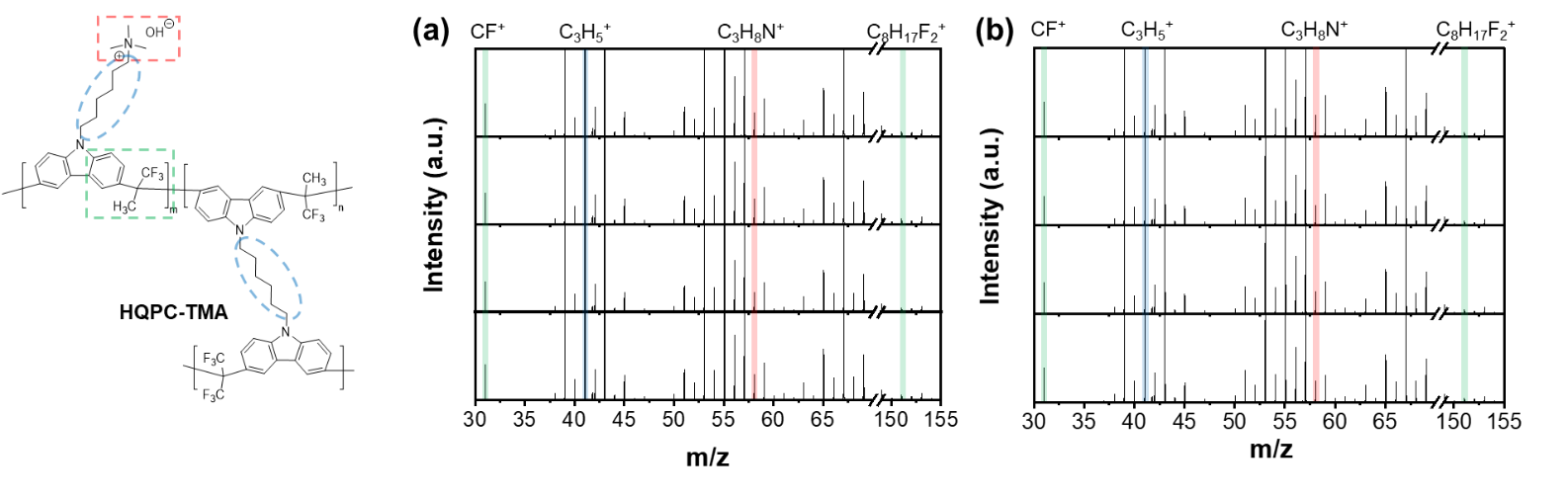


**Figure S34** ToF-SIMS profiles of HQPC-TMA membranes **(a)** before and **(b)** after the durability analysis.

|  | Waste alkaline water | |  | 1 M KOH | |
| --- | --- | --- | --- | --- | --- |
| Counts | **H_2_ (ml)** | **O_2_ (ml)** | Counts | **H_2_ (ml)** | **O_2_ (ml)** |
| 1^st^ | 3 | 1.5 | 1^st^ | 3 | 1.5 |
| 2^nd^ | 3 | 1.5 | 2^nd^ | 3 | 1.5 |
| 3^rd^ | 3 | 1.5 | 3^rd^ | 3 | 1.5 |
| 4^th^ | 3 | 1.5 | 4^th^ | 3.5 | 2 |
| 5^th^ | 3 | 2 | 5^th^ | 3 | 1.5 |
| Theoretical value  (at 400 mA) | **2.79** | **1.40** |  | **2.79** | **1.40** |

**Table S1.** The volume of produced H_2_ and O_2_ with theoretical value at -400 mA cm^-2^ analyzed through H-cell in half-cell test.

| Cathode | Anode | Electrolyte | Temperature  (^o^C) | Performance  (A cm^-2^, at 1.8 V) | Durability time  (h) | Degradation rate  (mV kh^-1^) | | | Ref. |
| --- | --- | --- | --- | --- | --- | --- | --- | --- | --- |
| NCC | **Co_3_O_4_** | **Waste alkaline water** | **60** | **0.45**  **(64 cm^2^)** | **2000** | | **50** | **This work** | |
|  |  |  |  | **0.6**  **(4.9 cm^2^)** |  |  |  |  |  |
| Mn-NiFe@WO_x_ | **Mn-NiFe@WO_x_** | **1 M KOH** | **70** | **0.88**  **(5 cm^2^)** | **300** | | **310** | | **[10]** |
| NiFeS  @Ti_3_C_2_ | **NiFeS**  **@Ti_3_C_2_** | **1 M KOH** | **50** | **0.27** | **35** | |  | | **[11]** |
| FeNi LDH | **CoP** | **1 M KOH** | **60** | **0.96**  **(5 cm^2^)** | **180** | |  | | **[12]** |
| VCoCO_x_@NF | **VCoC_x_@NF** | **1 M KOH** | **45** | **0.17** | **70** | |  | | **[13]** |
| NiMoO_x_@CMK-3 | **NiFe LDH** | **1 M KOH** | **50** | **0.46**  **(1 cm^2^)** | **400** | |  | | **[14]** |
| FeCoNiCuMo-HEA | **FeCoNiCuMo-HEA** | **1 M KOH** | **50** | **0.2** | **100** | |  | | **[15]** |
| NiMO_4_/MoO_2_ | **NiFe-BTC-GNPs** | **0.1 M KOH** | **70** | **0.44**  **(4 cm^2^)** | **120** | |  | | **[16]** |
| MoO_2_/Ni | **Ni Foam** | **1 M KOH** | **40** | **0.18**  **(78.5 cm^2^)** | **55** | | **7** | | **[17]** |
| Ni–W-600 | **NiFe LDH** | **1 M KOH** | **50** | **0.4**  **(5 cm^2^)** | **10** | |  | | **[18]** |

**Table S2.** The reference papers using non-precious metal for both cathode and anode.

Notes and references

[1] C.C. McCrory, S. Jung, J.C. Peters, T.F. Jaramillo, Benchmarking heterogeneous electrocatalysts for the oxygen evolution reaction, J Am Chem Soc, 135 (2013) 16977-16987.

[2] G. Kresse, J. Furthmüller, Efficient iterative schemes for ab initio total-energy calculations using a plane-wave basis set, Physical Review B, 54 (1996) 11169.

[3] P.E. Blochl, Projector augmented-wave method, Phys Rev B Condens Matter, 50 (1994) 17953-17979.

[4] B. Hammer, Improved adsorption energetics within density-functional theory using revised Perdew-Burke-Ernzerhof functionals, Phys Rev B, 59 (1999) 7413-7421.

[5] J.P. Perdew, K. Burke, M. Ernzerhof, Generalized Gradient Approximation Made Simple, PHYSICAL REVIEW LETTERS, 77 (1996) 3865-3868.

[6] M. Methfessel, A.T. Paxton, High-precision sampling for Brillouin-zone integration in metals, Phys Rev B Condens Matter, 40 (1989) 3616-3621.

[7] P.E. Blochl, O. Jepsen, O.K. Andersen, Improved tetrahedron method for Brillouin-zone integrations, Phys Rev B Condens Matter, 49 (1994) 16223-16233.

[8] P.E.B. Richard Dronskowski, Crystal Orbital Hamilton Populations (COHP). Energy-Resolved Visualization of Chemical Bonding in Solids Based on Density-Functional Calculations, J. Phys. Chem., 97 (1993) 8617-8624.

[9] V.L. Deringer, A.L. Tchougreeff, R. Dronskowski, Crystal orbital Hamilton population (COHP) analysis as projected from plane-wave basis sets, J Phys Chem A, 115 (2011) 5461-5466.

[10] S. Oh, H. Roh, H. Im, O. Seo, Y. Takagi, T. Watanabe, J. Woo Han, K. Yong, Mn doped hierarchical water splitting electrocatalyst: Synthesis, surface analysis and application to AEMWE, Chemical Engineering Journal, (2024).

[11] D. Chanda, K. Kannan, J. Gautam, M.M. Meshesha, S.G. Jang, V.A. Dinh, B.L. Yang, Effect of the interfacial electronic coupling of nickel-iron sulfide nanosheets with layer Ti3C2 MXenes as efficient bifunctional electrocatalysts for anion-exchange membrane water electrolysis, Applied Catalysis B: Environmental, 321 (2023).

[12] L. Wan, Z. Xu, B. Wang, Green preparation of highly alkali-resistant PTFE composite membranes for advanced alkaline water electrolysis, Chemical Engineering Journal, 426 (2021).

[13] A. Meena, P. Thangavel, A.S. Nissimagoudar, A. Narayan Singh, A. Jana, D. Sol Jeong, H. Im, K.S. Kim, Bifunctional oxovanadate doped cobalt carbonate for high-efficient overall water splitting in alkaline-anion-exchange-membrane water-electrolyzer, Chemical Engineering Journal, 430 (2022).

[14] Y. Chen, K. Yue, J.-W. Zhao, Z. Cai, X. Wang, Y. Yan, Effective modulating of the Mo dissolution and polymerization in Ni4Mo/NiMoO4 heterostructure via metal-metal oxide-support interaction for boosting H2 production, Chemical Engineering Journal, 466 (2023).

[15] P.-S. Jhu, C.-W. Chang, C.-C. Cheng, Y.-C. Ting, T.-Y. Lin, F.-Y. Yen, P.-W. Chen, S.-Y. Lu, Non-precious high entropy alloys and highly alkali-resistant composite membranes based high performance anion exchange membrane water electrolyzers, Nano Energy, 126 (2024).

[16] P. Thangavel, M. Ha, S. Kumaraguru, A. Meena, A.N. Singh, A.M. Harzandi, K.S. Kim, Graphene-nanoplatelets-supported NiFe-MOF: high-efficiency and ultra-stable oxygen electrodes for sustained alkaline anion exchange membrane water electrolysis, Energy & Environmental Science, 13 (2020) 3447-3458.

[17] F. Bartoli, L. Capozzoli, T. Peruzzolo, M. Marelli, C. Evangelisti, K. Bouzek, J. Hnát, G. Serrano, L. Poggini, K. Stojanovski, V. Briega-Martos, S. Cherevko, H.A. Miller, F. Vizza, Probing the activity and stability of MoO2 surface nanorod arrays for hydrogen evolution in an anion exchange membrane multi-cell water electrolysis stack, Journal of Materials Chemistry A, 11 (2023) 5789-5800.

[18] Y.K. Li, G. Zhang, H. Huang, W.T. Lu, F.F. Cao, Z.G. Shao, Ni(17) W(3) -W Interconnected Hybrid Prepared by Atmosphere- and Thermal-Induced Phase Separation for Efficient Electrocatalysis of Alkaline Hydrogen Evolution, Small, 16 (2020) e2005184.
